# Effectiveness of cognitive behavioural therapy-based interventions for maternal perinatal depression: a systematic review and meta-analysis

**DOI:** 10.1186/s12888-023-04547-9

**Published:** 2023-03-29

**Authors:** Danelle Pettman, Heather O’Mahen, Oscar Blomberg, Agneta Skoog Svanberg, Louise von Essen, Joanne Woodford

**Affiliations:** 1 Healthcare Sciences and e-Health, Department of Women’s and Children’s Health, Uppsala University, Uppsala, Sweden; 2 Mood Disorders Centre, Department of Psychology, University of Exeter, Exeter, UK; 3 Reproductive Health, Department of Women’s and Children’s Health, Uppsala University, Uppsala, Sweden

**Keywords:** Cognitive behavioral therapy, Perinatal, Postpartum, Postnatal, Pregnancy, Depression, Systematic review, Meta-analysis

## Abstract

**Background:**

Depression during the perinatal period (during pregnancy and the year after childbirth) is common and associated with a range of negative effects for mothers, infants, family members, and wider society. Although existing evidence suggests cognitive behavioral therapy (CBT) based interventions are effective for perinatal depression, less is known about the effect of CBT-based interventions on important secondary outcomes, and a number of potential clinical and methodological moderators have not been examined.

**Methods:**

A systematic review and meta-analysis primarily examined the effectiveness of CBT-based interventions for perinatal depression on symptoms of depression. Secondary aims examined the effectiveness of CBT-based interventions for perinatal depression on symptoms of anxiety, stress, parenting, perceived social support, and perceived parental competence; and explored clinical and methodological moderators potentially associated with effectiveness. A systematic search of electronic databases and other sources was performed up to November 2021. We included randomized controlled trials comparing CBT-based interventions for perinatal depression with control conditions allowing for the isolation of the effects of CBT.

**Results:**

In total, 31 studies (5291 participants) were included in the systematic review and 26 studies (4658 participants) were included in the meta-analysis. The overall effect size was medium (hedges *g* = − 0.53 [95% CI − 0.65 to − 0.40]); with high heterogeneity. Significant effects were also found for anxiety, individual stress, and perceived social support, however few studies examined secondary outcomes. Subgroup analysis identified type of control, type of CBT, and type of health professional as significant moderators of the main effect (symptoms of depression). Some concerns of risk of bias were present in the majority of studies and one study had a high risk of bias.

**Conclusions:**

CBT-based interventions for depression during the perinatal period appear effective, however results should be interpreted with caution given high levels of heterogeneity and low quality of included studies. There is a need to further investigate possibly important clinical moderators of effect, including the type of health professional delivering interventions. Further, results indicate a need to establish a minimum core data set to improve the consistency of secondary outcome collection across trials and to design and conduct trials with longer-term follow-up periods.

**Trial registration:**

CRD42020152254.

**Supplementary Information:**

The online version contains supplementary material available at 10.1186/s12888-023-04547-9.

## Introduction

Perinatal depression (PND) is a common mental health difficulty experienced during pregnancy and/or after childbirth, with global pooled prevalence rates estimated at 11.9% [1]. The impact of PND is significant for the mother, the infant, family members, and wider society. Negative effects for the mother include poor quality of life [2, 3], anxiety and stress [4, 5], and risk of death to the mother in the severest cases [6]. PND is also associated with difficulties in social relationships, partner relationships, and sexuality [6]. Critically, PND can have negative effects on the infant’s social, cognitive and emotional development, persisting into late childhood and adolescence [5]. This effect is mediated both directly via exposure to chronically elevated maternal cortisol during pregnancy, or indirectly, via the relationship between the parent and infant and parenting practices [7–11]. Mothers with PND are less likely to demonstrate sensitive and responsive interactions with their infants, and are more likely to report difficulties breastfeeding, establishing sleep routines, and attending vaccination appointments [12]. Psychological interventions, and investment in perinatal mental health services, are recommended for mothers with PND and other perinatal mental health disorders [13]. Importantly, mothers with PND report preferences for psychological support over medication, especially with concerns about the effects of medication on the infant [14]. However, despite these recommendations, gaps in mental health care provision in the perinatal period remain [15, 16].

Recent reviews have concluded that psychological interventions are probably effective for PND. However, some previous reviews have been broad in scope, for example including any type of psychological intervention (e.g., interpersonal psychotherapy (IPT), mindfulness and psycho education) [17, 18] or including both prevention and treatment interventions [19] and thus are highly heterogeneous [18]. Other reviews have been narrow in scope, for example focusing on interventions delivered in the postnatal period only [20, 21] or on specific delivery modalities (e.g., internet-administered interventions) [22, 23]. To the best of our knowledge, there is no recent systematic review and meta-analysis specifically focusing on the evidence-base for CBT-based interventions. Conducting a systematic review and meta-analysis focusing on CBT-based interventions may potentially reduce the high levels of heterogeneity present in more “broad scope” reviews [17–19]. Reducing high levels of clinical heterogeneity may also facilitate an exploration of a number of novel moderators, for example, the potential effect of the type of health professional delivering intervention and including parenting intervention components [24].

Additionally, there has been a large increase in the number of published randomized controlled treatment trials (RCTs) of CBT-based interventions for PND since the last review that focused on CBT-based interventions for PND was published [25]. Further, existing reviews of psychological interventions for PND have been criticised for being of low methodological quality and a need to conduct reviews of higher quality and avoid biases associated with cumulated evidence from individual trials of low methodological quality has been highlighted [17]. For example, existing reviews of CBT-based interventions for PND have included studies with non-randomized designs [25, 26], potentially resulting in biased estimations of effect [27].

Another limitation of the existing evidence base is that a number of important secondary outcomes have been largely unexamined [24], for example, anxiety, stress (individual and perceived parenting), parenting (e.g., sensitivity/responsiveness), perceived social support, and perceived parental competence. Given high comorbidity rates of PND and anxiety [4], the impact of stress on both mothers and the infant [28, 29], the association between PND and parenting difficulties [7], and poor social support [30], it is suggested that PND interventions should also try to improve these important outcomes. However, existing reviews have not adequately addressed the effect of CBT-based interventions on these secondary outcomes.

Finally, the increase in RCTs of CBT-based interventions for PND presents an opportunity to investigate a number of potential clinical and methodological moderators of intervention effectiveness. Whilst previous reviews [25] have examined some important clinical moderators (e.g., time point of intervention pregnancy/postnatal, and type of CBT intervention), there are a number of moderators potentially associated with effectiveness yet to be investigated. First, the potential moderating effect of the severity of depression at baseline has not been examined in reviews of CBT-based interventions for PND, despite evidence suggesting the effectiveness of CBT-based interventions for depression may vary by baseline severity [31]. Second, the relative effectiveness of the method of intervention delivery is important to investigate given recommendations that maternal mental health services should provide flexible and accessible intervention delivery formats to overcome multiple barriers to access experienced by mothers [32–34]. Third, little is known about the moderating effect of type of healthcare professional delivering the intervention (i.e., mental health provider or non-specialist provider). Given the global treatment gaps for PND, with up to 90% of mothers not receiving treatment in low- and middle-income countries (LMIC) [35], the provision of interventions by non-mental health specialist providers [36], may represent a solution to help close the treatment gap, should they be demonstrated to be effective. Finally, to date existing reviews of CBT-based interventions for PND have not examined the inclusion of parenting components as a moderator. Examining the potential effect of including intervention components targeting parenting is fundamental given the association between parenting difficulties, PND, and negative infant outcomes [7, 10, 11].

Given the aforementioned gaps in the current evidence base, an updated systematic review and meta-analysis of CBT-based interventions for PND is warranted. This review seeks to overcome the aforementioned limitations of previous reviews by: (1) attempting to reduce clinical heterogeneity by only including CBT-based interventions and excluding third wave CBT interventions and preventative interventions; (2) restricting study inclusion to RCTs whereby allocation and concealment procedures were determined to have a low risk of bias [24] and examine study quality as a potential moderator; and (3) investigating a number of clinical and methodological moderators potentially associated with effectiveness that are currently neglected in the literature.

The objectives are threefold:

1. To examine the effectiveness of CBT-based interventions for PND on symptoms of depression and depression diagnosis.
2. To examine the effectiveness of CBT-based interventions for PND on secondary outcome measures including: anxiety; stress (individual and perceived parenting stress); parenting (e.g., sensitivity/responsiveness); perceived social support; and perceived parental competence.
3. To investigate clinical and methodological moderators potentially associated with effectiveness.

## Method

The review protocol is published [24] and registered in PROSPERO (CRD 42020152254). Methods are informed by Cochrane guidance [37], the Centre for Reviews and Dissemination guidance [38], and reporting follows the PRISMA 2021 statement [39] (Additional file 1).

### Eligibility criteria

#### Population

Adult women (aged ≥16 years) with a diagnosis of PND, for example, Diagnostic and Statistical Manual of Mental Disorders (DSM) IV [40] or V [41] and/or reporting depression symptomatology within the perinatal period (from pregnancy to 12 months postnatal) using a validated tool (e.g., Edinburgh Postnatal Depression Scale (EPDS)) [42]. No limits were placed on depression severity given the variability in outcome measures and cut off scores across studies [43]. However, studies specifically designed to target populations referred to as “at risk” of depression were excluded.

#### Interventions

Eligible interventions explicitly targeted PND using CBT-based interventions, including standalone behavioral activation (BA) or problem-solving based interventions. CBT-based interventions were defined as interventions focusing on evaluating, challenging, and modifying dysfunctional beliefs [44], for example adopting treatment protocols in accordance with Beck’s manual [45]. Third wave CBT interventions such as mindfulness were excluded. Standalone BA interventions eligible for inclusion were defined as interventions targeting reductions in behavioral avoidance and increasing positively reinforcing activities [46], including pleasant activity scheduling [47, 48] and contextual BA models [49, 50]. Standalone problem-solving interventions eligible for inclusion were defined as interventions including a definition of personal problems, generation of multiple solutions to each problem, selection of the best solution, development of a systematic plan for this solution, and evaluation of the solution [51]. Eligible problem-solving intervention subtypes [44] included extended problem-solving therapy [52, 53], brief problem-solving therapy [54], and self-examination therapy [55].

No limitations were placed on the health professional group supporting or delivering the intervention, the clinical setting of intervention delivery, or method of intervention delivery. Following existing guidance [56, 57], self-help interventions were categorised as self-administered (no support provided in the use of the intervention), minimal contact (regular overview of materials in the provision of check-ins), and guided (regular support sessions provided to discuss progress and any process issues experienced using the materials). Interventions targeting a problem other than PND (e.g. bipolar affective disorder) or the prevention of PND in at-risk, but not currently symptomatic mothers were excluded.

#### Comparators

Eligible control conditions included: (1) no-treatment control; (2) wait-list control (WLC); (3) treatment-as-usual (TAU); (4) non-specific factors component control; (5) specific factors component control; and (6) active comparator, based on standard definitions [58]. Only trial designs allowing for the isolation of the effects of CBT were included [59].

#### Outcomes

Eligible studies used self-report or proxy/clinician administered standardized measures of depression or PND. Only studies using measures of depression with at least acceptable internal consistency (Cronbach’s alpha ≥0.70) and test-retest reliability (Cronbach’s alpha or correlation ≥0.70), as reported in outcome measurement validation studies, were included (Additional file 2). Secondary outcomes were self-report measures of: (1) anxiety; (2) individual stress; (3) perceived parental stress; (4) parenting (e.g., sensitivity/responsiveness); (5) perceived social support; and (6) parental competence. Observational parenting (e.g., sensitivity /responsiveness) measures (e.g., video tapes assessed with mind-mindedness coding manual (Meins & Fernyhough: Mind-mindedness coding manual, Version 2.2., unpublished) were also included.

#### Study designs

Only RCTs were included, with non-randomized and uncontrolled designs excluded. RCTs with randomization procedures explicitly not randomly allocated and/or with sequences not explicitly concealed (high risk of bias, in line with the Cochrane Collaboration’s Risk of Bias tool 2.0 (RoB 2.0)) [60] were excluded, in accordance with previous reviews, [57, 61] to minimize the risk of an inflated overall effect size resulting from the inclusion of low-quality studies [62].

### Literature search and study selection

#### Electronic searches

Eligible studies in English and Swedish were identified through a comprehensive electronic database search (ASSIA; CENTRAL; CINAHL; EMBASE; ISI Web of Science; MEDLINE; Prospero; PsycINFO; SCOPUS; and SweMed+), clinical trials registers (www.ClinicalTrials.gov and www.who.int/trialsearch/) and conference proceedings (BIOSIS Previews; Conference Proceedings Citation Index, Health Management Consortium and Web of Science with Conference Proceedings). Grey literature was identified using OpenGrey, ProQuest, and DiVA (publishing database for Scandinavian universities). Databases were searched using medical subject headings (MeSH) and text words in the title and abstract. An example of the search terms used are provided: (postpartum OR post-partum OR antepartum OR ante-partum OR partum OR prepartum OR pre-partum OR intrapartum OR intra-partum OR peripartum OR peri-partum OR postnatal OR post-natal OR perinatal OR peri-natal OR antenatal OR ante-natal OR prenatal OR pre-natal OR pregnant OR pregnancy OR pregnancies OR puerper* OR maternal OR trimester OR impregnated OR gravid* OR multigravid* OR primigravid* OR parity OR obstetric OR gestation OR “in utero” OR maternity OR partus OR obstetrical) AND (depression OR depressed OR depressive OR “low mood” OR mood OR distress OR wellbeing OR “well-being” OR emotion OR emotional OR melanchol* OR affect OR affective OR dysphori* OR dysthymia OR alexithymia) AND (cognitive OR behaviour OR behavioural OR behaviour OR behavioural OR cognitive behavio* OR “behavioural activation” OR “behavioral activation” OR “problem solving” OR ccbt OR icbt OR “cognitive restructuring” OR “cognitive reframing” OR “activity scheduling”) AND (therapy OR therapies OR psychotherapy OR intervention OR management OR “program evaluation” OR program OR programs OR programme OR programmes OR group OR course OR online OR internet OR web OR “web-based” OR phone OR telephone OR skype OR “e-therapy” OR etherapy OR “computer assisted” OR “internet intervention” OR computer OR computerised OR computerized OR mobile OR tablet OR smartphone OR “internet administered” OR “e-mental health” OR “m-mental health” OR Ehealth OR “e-health” OR “e-intervention”) AND (“randomized controlled trial” OR “randomized control trial” OR RCT OR controlled OR randomised OR randomized OR randomisation OR randomization OR “random assignment” OR “random allocation” OR random OR randomly OR control OR feasibility OR pilot OR “comparative study” OR “follow up” OR meta-analysis OR “meta analysis” OR review). The exact search terms used for each electronic database search can be found in Additional file 3. 

The search strategy was developed following PRESS Peer Review Guidelines [63] (Additional file 4). All databases were searched from inception until November 2021. Searches for relevant dissertations were conducted; however, full dissertations (Additional file 5) were not reviewed and studies identified in languages other than English and Swedish (Additional file 6) were not included due to time and funding limitations.

#### Hand searches

Forward citation searches were conducted using Google forward citation chasing [64] and reference lists were hand searched for all included studies. Studies identified in relevant secondary evidence reports (e.g. relevant systematic reviews and meta-analyses) were also reviewed. Study selection was managed using Endnote referencing management software (Version, 9) and Microsoft Access 2016. Study duplicates across electronic searches were removed. Disagreements regarding inclusion were discussed between two reviewers (DP & OB), with a third (JW) or fourth (HOM) reviewer consulted when needed to reach consensus. Two independent reviewers conducted a wide screen of study titles and abstracts, followed by full paper checks of potentially eligible studies. Studies were excluded if they did not clearly meet the outlined PICOS criteria (Additional file 7). Authors were contacted by email in the event of missing data, with a follow up email sent if there was no response within two weeks.

### Data extraction

Two reviewers independently extracted data from included studies and data was managed using Microsoft Excel 2016. Data extraction included: (1) study characteristics; (2) participant characteristics; (3) intervention characteristics; (4) study outcome measurements; and (5) participant flow. Discrepancies were discussed between the two reviewers (DP & OB), with a third reviewer (JW) consulted if consensus was not reached.

### Risk of bias assessment

Methodological quality of the primary studies was assessed using RoB 2.0 [60]. Reviewers assessed risk of bias independently across the following domains: (1) randomization; (2) allocation to intervention; (3) adherence to intervention; (4) handling of missing outcome data; (5) measurement of outcome; and (6) selection of the reported results. Overall risk of bias was rated as “low”, “some concerns”, and “high” for each domain both across and within studies. Ratings were compared, discrepancies discussed, and consensus reached with a third reviewer (JW) where necessary. Rate of retention was set at 80% (for the primary time point at or closest to 6 months) as opposed to 95% suggested by the tool, as a cut-off of 80% is recommended elsewhere to separate high and low quality RCTs [65].

### Statistical analysis

#### Measures of intervention effect

Meta-analysis was performed using Comprehensive Meta-Analysis version 3 [66]. Post-intervention between-group standardized mean effect sizes were calculated separately for primary (depression) and secondary outcomes (anxiety, individual stress, perceived parental stress, parenting, perceived social support, and parental competence) using Hedge’s g [67]. Incidence of major depressive disorder post-intervention was calculated using Odds Ratio (OR) alongside 95% confidence intervals (CIs) [68]. A primary end point ≤6 months post-intervention was adopted to minimise elevated effect sizes associated with short term follow up [69]. A random effects model [70] was adopted based on the expectation of large heterogeneity arising as a consequence of wide variations in the clinical and methodological parameters between studies [71].

Cochran’s Q statistic was used to examine the presence of heterogeneity [70], I^2^ was used to measure the proportion of total variability due to between-study heterogeneity and the prediction interval was used as an index of dispersion of the population [72]. I^2^ values are interpreted as low (above 25%), moderate (above 50%), and high (above 75%). On one occasion [73], a study included two CBT-based interventions delivered by different health professionals (nurses and psychologists), therefore comparisons were analysed separately, with the control condition sample size halved in each comparison. Where possible, intention-to-treat data was used, with completer data used when not available.

#### Sensitivity analysis

Sensitivity analyses for the overall effect size of the primary outcome measurement (depression) were conducted by temporary removal of: (1) each study individually from the overall analysis; (2) small studies (*n* ≤ 20 across conditions); and (3) studies with high attrition (≥30% in at least one arm), with the effect size recalculated.

#### Sources of possible bias

For outcome measures with at least 10 studies, funnel plot asymmetry was examined for sources of possible bias (e.g., publication bias, language bias, inclusion of small studies with poor methodological quality, and heterogeneity) [74, 75]. An estimated effect size taking biases into account was calculated using the trim and fill procedure [76].

#### Moderator analysis

Moderator analysis of associations between clinical and methodological moderators on the effect size for the primary outcome of depression were examined:

- Risk of bias (low vs. some concerns vs. high).
- Type of comparator (no-treatment control vs. WLC vs. TAU vs. non-specific factors component control vs. specific factors component control vs. active comparator).
- Length of follow up (short: post-intervention - less than 3 months vs. medium: 3–6 months vs. long: 7–11 months vs. extended:12 months+).
- Severity of depression at baseline (severe vs. moderate vs. mild), calculated using baseline mean scores and clinical cut offs for each depression measure.
- Type of CBT intervention (CBT vs. BA vs. problem solving).
- Interventions including additional social components (yes vs. no). Social components were defined as structured activities to improve social support e.g., partner session(s) or networking and communication skill building.
- Interventions including parenting intervention components (yes vs. no). Parenting intervention components were defined as including specific support in relation to the parent-infant relationship, for example specific sessions with a therapist, video feedback, or self-help materials (e.g., video interaction guidance) [77].
- Method of delivery (Individual ‘high intensity’ e.g., traditional CBT delivered by a trained psychological therapist workforce, typically weekly 60-minute sessions over at least a 10-week period [78] vs. group vs. guided or minimal contact self-help vs. self-administered self-help [56, 78, 79].
- Time point of intervention (prenatal vs. postnatal).
- Health professional delivering intervention (nursing professionals vs. social workers vs. psychologists vs. junior mental health workers vs. peers).

A random effects model was adopted, with *Q* reported as a measure of heterogeneity and I^2^ used to measure the proportion of total variability due to between-study heterogeneity [80]. Consistent with previous meta-analyses [56, 81], the alpha level was set at ≤.10 in the event of there being a low number of available comparisons with respect to moderator analyses. Under these circumstances differences are reported as a trend in the data.

### Protocol amendments

The following amendments were made to the published protocol: (1) the health professional delivering the interventions moderator was grouped into “non-specialist providers” e.g., peers and community workers, “health providers” e.g., nurses and midwives and “mental health providers” e.g., psychological wellbeing practitioners and clinical psychologists due to large variation in intervention providers; (2) a post-hoc moderator analysis was conducted comparing studies from LMIC and high income countries according to the World Bank classification [82]; and (3) the original protocol included a thematic synthesis of qualitative data to describe the acceptability of CBT-based PND interventions however due to the volume of qualitative studies eligible for inclusion being greater than anticipated, results are reported separately.

## Results

### Study selection

**Fig. 1:**
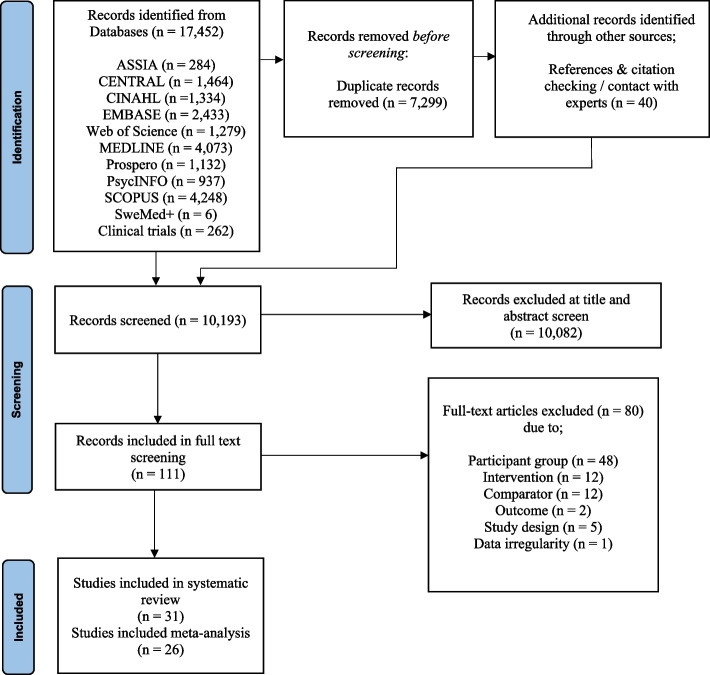
Prisma flow diagram of the inclusion of studies

A total of 17,452 studies were identified via electronic databases with 262 potential clinical trials identified through searching www.ClinicalTrials.gov and www.who.int/trialsearch/. A further 40 possible studies were identified through reference and citation checking and contact with experts in the field. Following duplicate removal, the search strategy yielded 10,193 records. A total of 111 full text articles were assessed for eligibility and of these, 31 studies were eligible and included in the narrative synthesis (Additional file 8) and 26 studies provided enough data for inclusion in the meta-analysis (see Fig. 1). For references to excluded studies, see Additional file 9 and for references to included studies, see Additional file 10.

### Study characteristics

A total of 5291 women were randomized in the 31 studies included in the narrative synthesis. Study characteristics are presented in Table 1 and intervention characteristics are presented in Table 2. Studies were conducted across 11 countries with 16.1% (5/31) conducted in LMICs. Thirty studies were published in academic journals and one was an unpublished report. The majority of studies provided information of funding sources, and ethical approval, with two studies providing no funding information [83, 84] and two providing no ethical information [85, 86].

**Table 1:** Study characteristics of studies included in the systematic review

Study	MDD Ax Yes/No (method)	Depression severity at baseline *m*	Participant ethnicity *n*, %	Sample ***n***	Recruitment setting	Control condition	Depression outcome measure, time points	Country	LMIC Country *m*
Alhusen et al. [87]	No	Moderate	African American 54, 90.0%	60	Clinical	TAU	EPDS, PI, 3 MFU	USA	No
			White 6, 10.0%						
Ammerman et al. [88]	Yes (SCID)	Severe	**Race**	93	Clinical	TAU	EPDS, PI, 3 MFU	USA	No
			White 58, 62.4%						
			African American 30, 32.3%						
			Native American 1, 1.1%						
			Native Hawaiian or other Pacific Islander 2, 2.2%						
			Bi-racial 2, 2.2%						
			**Ethnicity**						
			Latina 7, 7.5%						
			None 86, 92.5%						
Burns et al. [89]	Yes (CIS-R)	Moderate	**Ethnicity**	36	Clinical	TAU	EPDS, PI, 4 MFU	UK	No
			White 30, 83.3%						
Dimidjian et al. [90]	No	Moderate	**Race**	163	Clinical	TAU	PHQ-9, PI	USA	No
			White 95, 58.3%						
			Black 45, 27.6%						
			Asian 7, 4.3%						
			Other 16, 9.8%						
			**Hispanic ethnicity**						
			25, 15.3%						
Forsell et al. [91]	Yes (SCID)	Moderate	NI	42	Mixed	TAU	MADRS-S, PI	Sweden	No
Fuhr et al. [92]	No	Moderate	NI	280	Clinical	Enhanced TAU	PHQ-9, PI, 3 MFU	India	Yes
Honey et al. [85]	No	Moderate	NI	45	Clinical	TAU	EPDS, PI, 6 MFU	UK	No
Hughes et al. [32]	Yes (SCID) CO	Moderate	White 14, 20.0%	70	Clinical	TAU	EPDS, PI, 3 MFU	USA	No
			Black 10, 14.3%						
			Hispanic 41, 58.6%						
			Other 5, 7.1%						
Khamseh et al. [83]	No	Mild	NI	70	Clinical	TAU	BDI-II, PI, 1 MFU	Iran	Yes
Lund et al. [93]	Yes (MINI)	Mild	NI	425	Clinical	Enhanced TAU	HDRS, PI, 9 MFU	South Africa	Yes
McKee et al. [86]^a^	No	Moderate	Black 81, 43.3%	187	Clinical	TAU	BDI-II, PI	USA	No
			Hispanic 106, 56.7%						
Meager & Milgrom, [84]^a^	No	Severe	Australian born 16, 80.0%	20	Clinical	WLC	EPDS, PI	Australia	No
			From Ireland, Scotland and the United Kingdom 4, 20.0%						
Milgrom et al. [94]	Yes (CIDI) screening	Moderate	NI	192	Clinical	TAU	BDI-II, PI	Australia	No
Milgrom et al. [73]	No	Moderate	Born in Australia 56, 82.4%	68	Clinical	Enhanced TAU	BDI-II, PI	Australia	No
Milgrom et al. [95] A	Yes (SCID) screening	Severe	Born in Australia 42, 77.8%	54	Clinical	TAU	BDI-II, PI, 11 MFU	Australia	No
Milgrom et al., [96] B	Yes (CIDI) screening	Severe	NI	45	Clinical	AC	BDI-II, PI, 3 MFU	Australia	No
Milgrom et al., [97]	Yes (SCID)	Moderate	Born in Australia 39, 90.7%	43	Mixed	TAU	BDI-II, PI	Australia	No
Misri et al. [98]	No	Moderate	White 22, 62.9%	35	Clinical	AC	EPDS, PI	Canada	No
			South Asian 5, 14.3%						
			First Nations 3, 8.6%						
			Mexican 1, 2.9%						
			Spanish 1, 2.9%						
			Indo-Canadian 1, 2.9%						
			Italian 1, 2.9%						
			South American 1, 2.9%						
Morrell et al. [99]	No	Unknown	White British 390, 93.3%	418	Clinical	TAU	EPDS, PI	UK	No
Nasiri et al. [100]	No	Moderate	NI	120	Clinical	TAU	BDI-II, PI	Iran	Yes
Ngai et al. [101]^a^	No	NI	NI	397	Clinical	TAU	EPDS, PI, 6 MFU	Hong Kong	No
O’Mahen et al. [102]A	Yes (SCID) screening	Moderate	African American 32, 58.2%	55	Clinical	TAU	BDI-II, PI, 3 MFU	USA	No
			White 17, 30.9%						
			Asian 4, 7.3%						
			Other 2, 3.6%						
O’Mahen et al. [103]B	No	Severe	NI	910	Internet	TAU	EPDS, PI	UK	No
O’Mahen et al. [104]	Yes (CIS-R) screening	Severe	White/British 77, 92.8%	83	Internet	TAU	EPDS, PI, 6 MFU	UK	No
			Asian 1, 1.2%						
			Mixed white/African/Caribbean 2, 2.4%						
			African 1, 1.2%						
			Other 2, 2.4%						
Pugh et al. [105]	YES (MINI) screening only	Moderate	Caucasian 45, 95.7%	47	Mixed	WLC	EPDS, PI	Canada	No
			Other 2, 4.3%						
Rojas et al. [106]	YES (MINI) screening	Moderate	NI	230	Clinical	Enhanced TAU	EPDS, PI, 3 MFU	Chile	No
Sikander et al. [107]	No	Moderate	NI	570	Community	Enhanced TAU	PHQ-9, PI, 3 MFU	Pakistan	Yes
Trevillion et al. [108]	YES (SCID)	NI	White 35, 66.0%	53	Mixed	TAU	EPDS, PI, 3 MFU	UK	No
			Black: 14, 26.4%						
			Asian: 1, 1.9%						
			Mixed/Other: 3, 5.7%						
Van Lieshout et al. [109]	No	Moderate	White 291, 72.2%	403	Mixed	TAU	EPDS, PI	Canada	No
Wiklund et al. [110]^a^	No	Moderate	Born in Sweden 61, 91.0%	67	Clinical	TAU	EPDS, PI	Sweden	No
Wozney et al. [111]^a^	Yes (SCID)	Moderate	NI	62	Mixed	TAU	EPDS, PI, 6 & 12 MFU	Canada	No

*Abbreviations: AC* Active Control, *Ax* Assessment, *BDI-II* Beck Depression Inventory Second Edition, *CIDI* The Composite International Diagnostic Interview, *CIS-R* Clinical Interview Schedule-Revised, *EPDS* Edinburgh Postnatal Depression Scale; HDRS: Hamilton Depression Rating Scale, ICD-10: International Classification of Diseases 10; MADRS-S: Montgomery Åsberg Depression Rating Scale; MDD: Major Depressive Disorder; MFU: Month Follow Up; MINI: The Mini International Neuropsychiatric Interview; NI: No information; PHQ-9: Patient Health Questionniare-9; PI: Post Intervention; SCID: Structured Clinical Interview for DSM-IV; TAU: Treatment As Usual; UK: United Kingdom; USA: United States of America; WLC: Waiting List Control

^a^denotes included in systematic review only; *m* denotes moderator; & *n*: number of participants in the study

**Table 2:** Intervention characteristics of studies included in the systematic review

Study	Time point of intervention *m*	Type of CBT intervention *m*	Method of delivery *m*	No. of sessions/ modules	Health professional *m*	Social support *m*	Parenting componant *m*	Fidelity	Study specific training
Alhusen et al. [87]	Pre	CBT	Group	6	Mixed (SW & nurse)	Topic: social support systems, communication styles, and getting needs met	Incorporates attachment theory. Topics: stressors affecting mother-baby relationship and intergenerational transmission of thought patterns	Checklists and notes to ensure all content was covered	Training provided
Ammerman et al. [88]	Post	CBT	IHI	15 plus booster	MHP	No	Skills-based strategies used to increase maternal sensitivity to child cues	Checklists to ensure all content was covered. Supervision (weekly, provided by doctoral level clinicians)	NI
Burns et al. [89]	Pre	CBT	IHI	12	MHP	No	No	Sessions rated with CTS-R. Sessions recorded monitored for adherence (10%). Supervision (weekly)	Trained to deliver the intervention until judged to be competent by a PSY with specialist perinatal expertise
Dimidjian et al. [90]	Pre	BA	IHI	10	HP	No	No	Sessions rated with the QBAS. Role-plays rated by two BA experts	Training provided by authors included 2 days of in-person workshops and self-paced reading followed by ongoing weekly group telephonic supervision (90 min) and individual supervision (30 min).
Forsell et al. [91]	Pre	CBT	G/MC self-help	10 plus optional modules	MHP	No	No	NI	E-therapists had basic CBT training but no prior experience nor any special education or training in order to treat this specific population
Fuhr et al. [92]	Pre	BA	IHI	6 to 14	NSP	Collaboration with the family	No	Sessions rated with TQS. Group supervision (fortnightly, once a month with a supervisor present and once a month without a supervisor present)	25–40 h of classroom-based training. Comprised discussion and roleplays. A clinical internship period of 2 months followed the training. At the end of their training period competence was judged using standardized roleplays. Only those who passed predefined competence assessments were selected
Honey et al. [85]	Post	CBT	Group	8	HP	No	No	NI	NI
Hughes et al. [32]	Post	CBT	IHI	16	MHP	No	No	Supervision (weekly)	NI
Khamseh et al. [83]	Pre	PS	Group	5	Mixed (PSY nurse)	No	No	NI	NI
Lund et al. [93]	Pre	PS & BA	IHI	6	NSP	No	No	Checklists to ensure all content was covered. Supervision (weekly, group based with clinical social worker)	5 days of training by a clinical SW in basic counselling and delivery of the intervention
McKee et al. [86]^a^	Pre	CBT	IHI	8 CBT plus 4 parenting sessions	MHP	Social support building sessions. Unstructured opportunities for supportive companionship	Four child-development psychoeducational modules. Topics: sensitive and responsive mothering	NI	NI
Meager & Milgrom, [84]^a^	Post	CBT	Group	10	MHP	Partner session (n = 1). Group environment of social and emotional support	No	NI	NI
Milgrom et al. [94]	Post	CBT	Group	12 (3 with partner)	MHP	Partner sessions (*n* = 3)	No	Checklists to ensure all content was covered	One-to-one instruction in use of the therapy manuals and regular, intensive supervision from the principal investigator
Milgrom et al. [73]	Post	CBT	IHI	6	Mixed (PSY and nurse)	Partner session (n = 1)	No	NI	Half-day training workshop in the CBT intervention. The training was conducted by a senior PSY with several years experience in delivering CBT for postnatal depression
Milgrom et al. [95] A	Pre	CBT	IHI	8	MHP	Partner session (n = 1)	No	Checklists and notes to ensure all content was covered	Training provided for pregnancy-specific CBT programme
Milgrom et al. [96] B	Post	CBT	Group	12	MHP	Partner sessions (n = 3)	No	Checklists to ensure all content was covered	NI
Milgrom et al., [97]	Post	CBT	G/MC self-help	6	MHP	Access to literature for partner	No	NI	Training included working through the program (as if they were a participant), reading the coach manual, observing other coaches’ complete calls, and a verbal explanation from a senior PSY about the role and the tasks involved
Misri et al. [98]	Post	CBT	IHI	12	MHP	No	No	NI	NI
Morrell et al. [99]	Post	CBT	IHI	8	HP	No	No	NI	Trained to deliver psychologically informed sessions based on cognitive behavioral principles
Nasiri et al. [100]	Post	PS	IHI	6	Mixed (Midwife & PSY)	No	No	NI	A clinical psychologist supervised performance on the first 10 participants
Ngai et al. [101]^a^	Post	CBT	IHI	5	HP	No	No	Supervision (bi-weekly and tape review (10%) by the research team)	20 hours of CBT training
O’Mahen et al. [101]A	Pre	CBT	IHI	12	MHP	No	No	Sessions rated with CTS-R. Supervision (weekly and tape review (10%) by clinical supervisor)	Training consisted of: reading the manual, review and training in key concepts with either the principal investigator or clinical supervisor and co-investigator, and completion of an initial participant under close supervision
O’Mahen et al. [103]B	Post	BA	Unguided self help	11	n/a	Topic: addressing support with new mother. Netmums ‘meet a mum’ feature, to connect with other women in their local area	No	NI	n/a
O’Mahen et al. [104]	Post	BA	G/MC self-help	12	MHP	Netmums ‘meet a mum’ feature, to connect with other women in their local area	No	Supervision (weekly and tape review (20%) with chief investigator)	5 days of training in the ‘high-intensity’ (functional analysis-based) perinatal-specific BA approach. Training involved a mix of didactics and roleplay around conducting functional analysis in perinatal-specific domains with the chief investigator, a clinical PSY with specialty expertise in BA and perinatal depression, and an IAPT trainer
Pugh et al. [105]	Post	CBT	G/MC self-help	7	MHP	No	No	NI	A training workshop
Rojas et al. [106]	Post	CBT	Group	8	HP	No	No	Supervision (weekly)	8 hours of training
Sikander et al. [107]	Pre	BA	IHI	14	NSP	Collaboration with the family	No	Sessions rated with ENACT rating scale. Supervision (group)	Brief classroom training and regular group training. Field supervision by local trainers who were not mental health specialists, and these trainers were supervised by a specialist therapist, generating a cascade model of training and supervision
Trevillion et al. [108]	Pre	CBT	G/MC self-help	8	MHP	No	No	Checklists to ensure all content was covered. Sessions rated with CTS-R. Randomly selected tape review (20%) by PSY	Trained to deliver the intervention
Van Lieshout et al. [109]	Post	CBT	Group	1	MHP	Section on getting support from others	No	Practitioners observed delivering trial workshops prior to RCT	1 day classroom training
Wiklund et al. [110]^a^	Post	CBT	IHI	NI	HP	No	No	NI	NI
Wozney et al. [111]^a^	Post	CBT	G/MC self-help	12 plus booster	NSP	Partner/companion information brochure	No	Supervision (weekly) with an expert clinician	Training involved reading the handbook, observing others complete calls and verbal modelling and explanations from a senior clinician about the role and the tasks involved

*Abbreviations: BA* Behavioral Activation, *CBT* Cognitive Behavioral Therapy, *CTS-R* Cognitive Therapy Scale Revised, *CO* Control Only, *ENACT* Enhancing Assessment of Common Therapeutic factors, *G/MC self-help* Guided/Minimal Contact Self-Help, *HP* Health Provider, *IHI* Individual High Intensity, *IAPT *Improving Access to Psychological Therapies programme, *Mixed* Mixed provider, *MHP* Mental Health Provider, *NI* No information, *NSP* Non-specialist provider, *Pre* Prenatal, *Post* Postnatal, *PS* Problem Solving, *PSY* Psychologist, *QBAS* Quality of Behavioral Activation Scale, *SA* self-help: Self-administered self-help, *SW* Social Worker; *TQS* Therapy Quality Scale

^a^denotes included in systematic review only; *m* denotes moderator; & *n*: number of participants in the study

### Participants

In total, 38,059 women were screened across the 31 included studies with 5291 randomised, yielding an overall inclusion rate of 13.9% (5291/38059). Women’s mean age was 28.5 (SD 6.0, range 16–42 years) across the 25 studies reporting mean age. Interventions started during pregnancy in 12 studies, and during the postnatal period in 19 studies. In 15 studies, women were required to meet diagnostic criteria for MDD to be included, as measured by either the Structured Clinical Interview for DSM-IV (SCID; 8 studies); the Mini International Neuropsychiatric Interview (MINI; 3 studies); the Clinical Interview Schedule-Revised (CIS-R; 2 studies); or the Composite International Diagnostic Interview (CIDI; 2 studies). In the remaining 16 studies women needed to meet a standardised clinical cut off on a validated self-report measure of depression, including the EPDS (10 studies, cut offs ranging from ≥10 to ≥13) the BDI-II (3 studies, cut offs ranging from ≥11 and ≥ 14), and the PHQ-9 (3 studies, cut off ≥10) to be included.

Included studies described the cultural identities of women in diverse ways, with studies reporting ethnicity, race, or in some cases country of birth (Table 1). Average household income was reported using different currencies and was not suitable for synthesis, however three studies targeted low-income women [86, 106, 112], with one study specifically targeting minority ethnic women with low incomes [86]. In total, 28 studies reported severity of depression at baseline, with overall severity mild in two studies, moderate in 20 studies, and severe in six studies (Additional files 11 and 12).

### Intervention

In the 31 studies, 32 CBT-based interventions were compared with a control group. Intervention characteristics of studies included in the systematic review are presented in Table 2. The majority of the interventions were based on CBT (24 interventions), others were labelled as standalone BA (5 interventions), standalone problem solving (2 interventions), or labelled as combined BA and problem solving (1 intervention). Methods of delivery included individual high intensity (17 interventions), group (8 interventions), guided or minimal contact self-help (6 interventions), and self-administered self-help (1 intervention). In the 25 interventions delivered in person/via the telephone, the number of sessions ranged from five to sixteen. In group interventions, group sizes ranged from 4 to 20 over 1–12 sessions. In total, five self-help interventions were delivered online and two were delivered by a workbook. Social support components were included in 12 interventions and parenting components included in two interventions.

Thirty-one interventions were supported or delivered by a variety of providers including health care providers such as nurses (8 interventions), mental health providers such as clinical psychologists, psychological therapists, and psychological practitioners (18 interventions), non-specialist providers such as peers (4 interventions) and mixed providers, consisting of health and mental health providers (2 interventions). Twenty-two interventions were delivered in person in a range of settings including in clinics (16 interventions), home (4 interventions), and mixed clinic/home settings (2 interventions).

### Meta-analysis results for primary outcomes

**Fig. 2:**
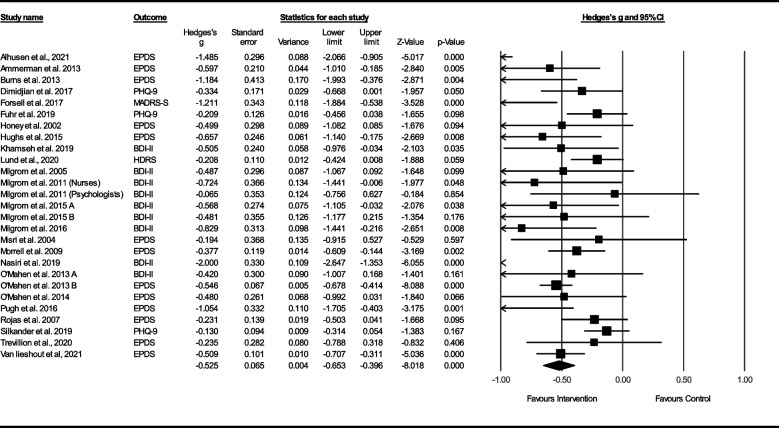
Effect sizes (Hedges g) and 95% confidence intervals for depression with time point of ≤6 months post-treatment

CBT-based interventions for PND resulted in a medium effect size, Hedge’s g = − 0.53 (95% CI = − 0.65 to − 0.40; z = − 8.02; *p* < .001) for depression symptoms (26 studies, 27 comparisons, *n* = 4658) using a random effects model. Effect sizes and 95% CIs of the studies are shown in Fig. 2. Estimates of between-study variance were high and statistically significant (p < .001, Q = 77.0) and *I*^2^ = 66.25. High heterogeneity is reflected in the prediction intervals, which indicated that the true effect size falls in the interval − 1.05 to 0.00 (Additional file 13).

#### Sensitivity analyses

Sensitivity analyses revealed no significant change in effect size or *p*-value when temporarily removing each study from the analysis. Sensitivity analysis for studies with a small sample size was not possible as no studies met the a priori criteria of *n* ≤ 20 across conditions. In the 25 studies reporting attrition data, 9 studies (10 comparisons) with high attrition rate (≥ 30% in at least one arm) yielded a medium effect size, Hedge’s g = − 0.67 (95% CI = − 0.94 to − 0.41) and 15 studies (15 comparisons) with a low attrition rate yielded a small effect size, Hedge’s g = − 0.49; (95% CI = − 0.69 to − 0.32). Post-hoc analysis of the 9 studies (10 comparisons) with high attrition, revealed that the four studies using completer data only, yielded a large effect size, Hedge’s g = − 1.01 (95% CI = − 1.74 to − 0.26) and five studies (6 comparisons) using imputed data yielded a medium effect size, Hedge’s g = − 0.54 (95% CI = − 0.66 to − 0.42).

#### Sources of possible bias

The funnel plot and Egger’s test did not suggest significant publication bias and the trim and fill procedure suggested 0 studies were missing to the right of the mean effect, with an imputed point estimate Hedge’s g = − 0.44; (95% CI = − 0.51 to − 0.38), (Additional file 14).

#### Moderator analysis

Moderator analysis revealed three significant moderators on the overall effect size for depression; type of control [Q = 26.44, df = 3, *p* < .001], type of CBT intervention [Q = 9.50, df = 3, *p* = 0.02], and type of health professional delivering the intervention [Q = 23.19, df = 3, *p* < .001] (see Table 3). With respect to type of control, studies with a WLC or TAU yielded significantly larger effect sizes (*p* = < .001) than those using an active control or enhanced TAU. Studies using problem solving or CBT yielded significantly larger effects sizes (*p* = 0.02) than studies using BA or BA plus problem solving. Studies with interventions delivered by mental health providers and mixed providers (e.g., mental health provider and/or a health provider) yielded significantly larger effect sizes (*p* = < .001) than interventions delivered by only health or non-specialist providers. A trend was found for length of follow up (*p* = 0.09), with a short length of follow up yielding larger effect sizes than studies with medium or long follow ups. The remaining moderators including: risk of bias; severity of depression at baseline; inclusion of social components; inclusion of parenting components; method of delivery; point of intervention; and LMIC setting were all non-significant.

**Table 3:** Moderator analysis

Moderators	No of comparisons	Hedges’ g	95%CI	Q Between	***P*** value	***I2***
**Risk of bias**	27	−0.5	−0.62 to −0.38	1.45	0.23	
High	1	−0.38	−0.61 to −0.14			0
Some concerns	26	−0.54	−0.68 to −0.41			66.46
**Type of control**	27	−0.37	−0.45 to −0.28	24.74	< .001*	
Active control	2	−0.34	−0.84 to 0.16			0
Enhanced TAU	6	−0.21	−0.32 to −0.10			0
TAU	18	−0.64	−0.80 to −0.49			59.41
WLC	1	−1.05	−1.71 to −0.40			0
**Length of follow up**	27	−0.47	−0.58 to −0.36	4.75	0.09	
Long	2	−0.3	−0.52 to −0.09			0
Medium	13	−0.47	−0.65 to −0.29			62.96
Short	12	−0.64	−0.86 to −0.42			66.62
**Severity of depression at baseline**	25	−0.52	−0.62 to −0.43	4.59	0.1	
Mild	2	−0.28	−0.53 to −0.03			21.11
Moderate	18	−0.62	−0.82 to −0.41			75.10
Severe	5	−0.55	−0.66 to −0.43			0
**Type of CBT intervention**	27	−0.43	−0.53 to −0.32	9.50	0.02*	
BA	5	−0.33	−0.54 to −0.21			73.63
CBT	19	−0.57	−0.71 to −0.42			41.98
PS	2	−1.24	−2.70 to 0.23			92.53
PS & BA	1	−0.21	−0.42 to −0.01			0
**Interventions including social components**	27	−0.53	−0.66 to −0.40	0.39	0.53	
No	15	−0.58	−0.77 to −0.38			68.06
Yes	12	−0.49	−0.67 to −0.31			66.87
**Interventions including parenting components**	27	−0.49	−0.62 to −0.37	1.4	0.24	
No	25	−0.48	−0.61 to −0.36			61.04
Yes	2	−1.02	−1.89 to −0.15			83.28
**Method of delivery**	27	−0.54	−0.64 to −0.44	1.66	0.65	
Group	7	−0.55	−0.80 to −0.30			59.53
Individual High Intensity	14	−0.47	−0.66 to −0.28			69.61
Guided/minimal contact self-help	5	−0.72	−1.08 to −0.37			42.44
Self-administered self-help	1	−0.55	−0.68 to −0.41			0
**Point of intervention**	27	−0.53	−0.66 to −0.41	0.31	0.58	
Prenatal	11	−0.48	−0.70 to −0.27			54.33
Postnatal	15	−0.57	−0.74 to −0.40			70.67
**Professional delivering intervention**	26	−0.37	−0.44 to −0.29	23.19	< .001*	
Health provider	5	−0.35	−0.50 to −0.20			0
Mental health provider	15	−0.56	−0.69 to −0.43			0
Mixed providers	3	−1.31	−2.20 to −0.42			86.76
Non-specialist providers	3	−0.17	−0.30 to −0.05			0
**Post hoc moderator**						
**Low middle income country**	27	−0.53	−0.64 to −0.42	0.05	0.82	
Yes	5	−0.49	−0.85 to −0.13			35.15
No	22	−0.53	−0.65 to −0.42			87.13

*Abbreviations*: *BA* Behavioral Activation, *CBT* Cognitive Behavioral Therapy, *n* number of comparisons, *TAU* Treatment as usual, *PS* Problem solving, *WLC* Waiting list control

#### Risk of bias

The majority of studies were rated as “some concern” of risk of bias (25 studies) and one study had a “high” risk of bias (Additional file 15).

### Meta-analysis results for secondary outcomes

Meta-analysis for secondary outcomes where data was available (13 studies, 14 comparisons, *n* = 1689) are reported in Table 4.

**Table 4:** Meta-analysis for secondary outcomes

Outcome	No of studies	Random effects				Heterogeneity			
		Hedges g	95% CI	Z	***P*** value	***P*** value	***Q Between***	***I***^**2**^	Prediction Intervals
Anxiety	14	−0.44	−0.55 to −0.33	−7.76	< .001	0.77	9.02	0.000	All studies share a common effect size
Individual stress	5	−0.56	−0.80 to −0.32	−4.49	< .001	< .001	2.99	0.001	All studies share a common effect size
Perceived parental stress	4	−0.16	−0.77 to 0.45	−0.51	0.61	< .001	21.33	85.80	−3.66 to 3.34
Self-report parenting	4	0.94	−0.01 to 1.88	1.96	0.05	< .001	43.69	93.13	−3.61 to 5.49
Perceived social support	6	0.25	0.14 to 0.36	4.46	< .001	0.32	5.88	14.95	−0.41 to 0.91

#### Anxiety

A small effect size was found for anxiety, Hedge’s g = − 0.44 (95% CI = − 0.55 to − 0.33; z = − 7.76; *p* < .001). Analysis of heterogeneity was non-significant (*p* = 0.77, Q = 9.02, *I*^2^ = 0.00). Trim and fill analysis suggested 0 studies were missing to the right of the mean effect, with an imputed point estimate Hedge’s g = − 0.44; (95% CI = − 0.55 to − 0.33) (Additional file 16).

#### Stress and social support

A medium effect size was found for individual stress, Hedge’s g = − 0.56 (95% CI = − 0.80 to − 0.32; z = − 4.49; *p* < .001). Analysis of heterogeneity was not significant [*p* < .001, *Q* = 2.99, *I*^2^ =0.00]. A small effect size was found for social support, Hedge’s g = 0.25 (95% CI = 0.14 to 0.36; z = − 4.46; *p* < .001). Analysis of heterogeneity was not significant (*p* = 0.32, Q = 5.88, *I*^2^ = 14.95).

#### Parenting

No significant effects were found for perceived parental stress, Hedge’s g = − 0.16 (95% CI = − 0.77 to 0.45; z = − 0.51; *p* = 0.61). A large effect size was found for self-reported parenting, Hedge’s g = 0.94 (95% CI = − 0.01 to 1.88; z = 1.96; *p* = 0.05). Only one study reported outcomes for parental competence with a large effect size for CBT-based interventions for perinatal depression on parental competence Hedge’s g = 0.87; (95% CI = 0.26, 1.49; z = 2.78; *p* < 0.05) [86]. No studies reported observational parenting outcome measures.

#### Incidence of major depressive disorder

Five studies measured incidence of major depressive disorder post intervention, yielding a significant effect, OR = 0.21 (95% CI = 0.07 to 0.61, Z = -2.90, *p* < .05).

## Discussion

Results of this systematic review and meta-analysis provide some support for the effectiveness of CBT-based interventions for PND, are in line with previous meta-analyses in the area [17, 18, 25], and findings remained significant after sensitivity analyses. Notably, effects were present in study populations that included women from a range of cultural and socio-economic backgrounds, including LMIC settings. However, studies with higher rates of attrition yielded significantly larger effect sizes than studies with smaller rates of attrition, especially when examining completer data only. There was a trend for studies with a shorter length of follow up to yield larger effect sizes than studies with medium or long-term follow ups. Only a small number of studies included important secondary outcome measures. Despite limiting the meta-analysis to studies of CBT-based interventions for PND, heterogeneity was high and similar to meta-analyses including a range of psychological interventions for the population [18], suggesting caution in interpreting these findings.

Moderator analysis indicated several significant moderators that may warrant further investigation. Consistent with previous research, studies with a WLC yielded significantly larger effect sizes than other control conditions [113–115]. Studies adopting a TAU control condition also yielded significantly larger effect sizes than those adopting an active control or enhanced TAU. Importantly, TAU has been identified as highly heterogeneous, with the effects of psychological interventions for adult depression found to differ, especially across countries [116]. Present results may indicate that perinatal women may not be receiving appropriate mental health treatment within usual care settings, a finding consistent with research demonstrating that access to evidence-based mental health treatment for women in the perinatal period is low, for example, within the United States, up to 85% of women with PND are estimated to remain untreated [117] and up to 90% of mothers in low- and middle-income countries do not receive treatment [35].

Type of professional delivering/supporting the intervention was also a significant moderator, with higher effect sizes found when the intervention was delivered by mental health and mixed providers (e.g., mental health provider and health provider teams) than interventions delivered only by health providers (e.g., midwives, health visitors, community nurse) or non-specialist providers (e.g., peer supporters). To the best of our knowledge, the moderating effect of type of healthcare professional delivering/supporting the intervention has not been previously examined within a PND population, and these findings require further investigation in future trials. Other research suggests guidance provided by certified psychotherapists versus general practitioners or medical doctors specialised in mental health may be associated with higher levels of adherence to internet-administered CBT for adult depression, but not overall symptom reduction [118]. Conversely, present findings indicate a need for interventions to be delivered by mental health providers, or within mixed health and mental health provider teams. There is a need for future research examining the impact of managing competing professional roles and identities [119, 120] on PND treatment delivery and what barriers health professionals may experience delivering perinatal mental health interventions [121, 122]. Future research may also wish to examine how PND treatment fidelity [123] may vary across professional groups to further inform training and supervision. Finally, a small effect size for non-specialist providers, indicates a need for enhancing training and supervision provided to develop and maintain competencies in line with the new competency framework for peer support workers [124].

Interestingly, type of CBT intervention was also a significant moderator, with larger effect sizes associated with CBT and problem-solving interventions versus BA interventions. Although this is a departure from previous meta-analyses of CBT intervention types for depression both within [125] and outside the perinatal period [126], it is important to note that three of the five BA studies included were delivered by health or non-specialist providers, suggesting intervention type and type of provider may have been confounded. Finally, we found a trend for higher depression severity at baseline to be associated with larger effect sizes. These results provide reassurance to clinicians treating women with more severe symptoms of depression and are consistent with reviews for adult depression [127].

Importantly, method of delivery was not a significant moderator of effect, and is supported by a mounting body of evidence from meta-analytic studies showing that method of delivery does not affect treatment effectiveness [18, 25, 79]. Similarly, there were no differences in the effect of the intervention on depression symptoms based on point of intervention, suggesting that interventions delivered either during pregnancy or postnatally are effective. We also found no evidence to suggest the inclusion of social or parenting interventions components were associated with effectiveness. However, it is important to note that in the 12 studies including some form of social component, components ranged from the provision of an informational brochure for a family member, work on communication skills, to including a family member in part of the treatment. Additionally, only two studies included parenting components, and given the association between parenting difficulties, PND, and negative infant outcomes [7, 10, 11] initial findings point strongly to the need for more research investigating CBT-based interventions that incorporate parenting components [11].

Finally, only a minority of studies in this review measured secondary outcomes and there was a lack of consistency regarding secondary outcomes adopted across trials. CBT-based interventions for PND produced large effects on self-reported parenting and parenting competence, moderate effect sizes on individual stress, and small effects on anxiety symptoms and perceived social support. No significant effects were found on perceived parental stress outcomes. Future CBT-based perinatal depression interventions would benefit from proactively addressing the problem of secondary outcome measurement, for example through the development of a minimum core data set for studies of interventions for perinatal depression [128].

### Strengths and limitations

Strengths include: (1) literature search and study selection, data extraction, risk of bias assessment was completed by two independent reviewers; (2) a comprehensive search strategy, including electronic databases, grey literature, clinical trial registers, hand searching, and following PRESS Peer Review Guidelines [63] was conducted; (3) excluding RCTs with high risk of bias concerning randomization procedures, may have reduced the risk of an inflated effect size and increased the methodological quality of included studies; and (4) examination of important secondary outcomes and methodological and clinical moderators.

Limitations include: (1) limiting studies to those in English or Swedish which may have introduced language bias; (2) the small number of studies included in the moderator analysis means analysis was underpowered [129] and provides only correlational data with the potential for confounding between moderator variables [130]; (3) the methodological quality of included studies was low, with 96% of studies reporting “some concerns” and one study reporting a “high” risk of bias, however, the RoB 2.0 may not be suitable for psychotherapy outcome research [131] given challenges associated with blinding of participants and treatment providers [132]; (5) the potential dependency of effects was managed using a simplistic approach [133] and analysed potentially dependent effects as if they were non-independent, increasing the risk of Type I errors [134], an adoption of a three-level meta-analytic model may have been more appropriate [134, 135]; and (6) due to heterogeneity in the depression outcome measures adopted by included studies, severity of depression at baseline (severe vs. moderate vs. mild) was calculated using baseline mean scores and clinical cut offs for each depression measure, with moderator analysis performed. However, using mean depression scores to calculate severity of depression at baseline may reduce the variance of severity included in the meta-analysis, limiting our ability to make more definitive conclusions concerning the potential moderating effect of the severity of depression at baseline. The moderating effect of severity of depression at baseline could be further examined by conducting an individual patient data meta-analyses and examining the interaction between baseline severity and treatment effect using multilevel linear regression.

## Conclusion

Findings from this meta-analysis demonstrate that CBT-based interventions for perinatal depression are effective both during pregnancy and the postnatal period for symptoms of depression. However, results should be interpreted with caution given high levels of heterogeneity and low quality of included studies. Results indicated that whilst it is important to increase access to PND interventions, caution should be exercised when utilizing healthcare and non-specialist providers without proper mechanisms in place to facilitate training, enhance fidelity, and avoid reduction of the power of interventions. Further, there is a need to conduct future research to examining factors such as the role of training, supervision, and treatment fidelity on treatment outcomes across different healthcare providers. Findings also highlight a need to integrate evidence-based parenting components into CBT-based interventions for PND, to establish a minimum core data set to improve the consistency of secondary outcome collection across trials, and conduct trials with longer-term follow-up periods.

## Supplementary Information

**Additional file 1.** PRISMA checklist. 

**Additional file 2.** Quality of included primary outcome measurements.

**Additional file 3.** Electronic literature search strategies.

**Additional file 4.** Peer review assessment form.

**Additional file 5.** References to papers in languages other than English or Swedish.

**Additional file 6.** References to dissertations.

**Additional file 7.** PICOS statement.

**Additional file 8.** Full paper PICOS screening.

**Additional file 9.** References for excluded studies.

**Additional file 10.** References for included studies.

**Additional file 11.** Severity of depression calculation.

**Additional file 12.** Depression outcome cut offs.

**Additional file 13.** Prediction interval.

**Additional file 14.** Funnel plot for depression.

**Additional file 15.** Funnel plot for anxiety.

**Additional file 16.** Risk of bias assessment.

## Data Availability

The datasets generated and analysed during the current study are available in the zendo repository, https://zenodo.org/record/6660969#.Yq43fqLMKUd.

## Abbreviations

BA: Behavioural Activation
CBT: Cognitive Behavioural Therapy
LMIC: Low Middle Income Country
MDD: Major Depressive Disorder
MeSH: Medical Subject Headings
NSP: Non-specialist Provider
RCT: Randomised Controlled Trial
TAU: Treatment as Usual
WLC: Wait List Control
