# Supplementary material for: Effectiveness of cognitive behavioural therapy-based interventions for maternal perinatal depression: a systematic review and meta-analysis
Source: BMC Psychiatry. 2023 Mar 29;23:208. doi: 10.1186/s12888-023-04547-9 (PMC10052839; doi:10.1186/s12888-023-04547-9)
Supplement: Supplementary file 2 — Additional file 2. Quality of included primary outcome measurements. [file 12888_2023_4547_MOESM2_ESM.docx]

S2. Quality of included primary outcome measurements

| **Outcome measurement** | **Number of studies** | **Internal consistency** (Cronbach's alpha) | **Test-retest reliability**  (Cronbach's alpha / correlation) |
| --- | --- | --- | --- |
| EPDS | 17 | 0.85  (Martin & Redshaw, 2018) | 0.92  (Kernot, Olds, Lewis & Maher, 2014 ) |
| BDI-II | 9 | 0.92  (Beck, Steer & Brown, 1996) | 0.93  (Beck, Steer & Brown, 1996) |
| PHQ-9 | 3 | 0.89  (Kroenke, 2001) | 0.84  (Kroenke, 2001) |
| HDRS | 1 | 0.75  (Luckenbaugh et al., 2015) | 0.74  (Trajkovic et al., 2011) |
| MADRS-S | 1 | 0.84  (Fantino, 2009) | 0.78  (Fantino, 2009) |

*Note.* Outcome measurement: EPDS = Edinburgh Postnatal Depression Scale; BDI-II = Beck Depression Inventory 2nd edition; HDRS: Hamilton Depression Rating Scale; PHQ-9 = Patient Health Questionnaire; & MADRS-S: Montgomery Åsberg Depression Rating Scale

**References**

Beck, A. T., Steer, R. A., Brown, G. K. (1996). BDI-II: Beck Depression Inventory Manual (2nd ed). *San Antonio: Psychological Corporation.*

Fantino, B., & Moore, N. (2009). The self-reported Montgomery-Åsberg depression rating scale is a useful evaluative tool in major depressive disorder. *BMC Psychiatry, 9*, 26.  https://doi.org/[10.1186/1471-244X-9-26](https://dx.doi.org/10.1186%2F1471-244X-9-26)

Kernot, J., Olds, T., Lewis, L. K., & Maher, C. (2015). Test-retest reliability of the English version of the Edinburgh Postnatal Depression Scale. *Archives of Women’s Mental Health, 18*(2), 255–257.<https://doi.org/10.1007/s00737-014-0461-4>

Kroenke, K., Spitzer, R. L., & Williams, J. B. W. (2001). The PHQ-9: Validity of a brief depression severity measure. *Journal of General Internal Medicine, 16*(9), 606–613. <https://doi.org/10.1046/j.1525-1497.2001.016009606.x>

Luckenbaugh, D.A., Ameli, R., Brutschem N.E. & Zarate, C.A. (2015). Rating depression over brief time intervals with the Hamilton Depression Rating Scale: Standard vs. abbreviated scales*. Journal of Psychiartic Research*, 61. <https://doi.org/10.1016/j.jpsychires.2014.12.015>

Martin, C. R., & Redshaw, M. (2018). Establishing a coherent and replicable measurement model of the Edinburgh Postnatal Depression Scale. *Psychiatry Research, 264*, 182–191.

https://doi.org/10.1016/j.psychres.2018.03.062

Trajkovic, G., Starcevic, V., Latas, M., Lestarevic, M., Ille, T., Bukumiric, Z. & Marinkovic, J. (2011). Reliability of the Hamilton Rating Scale for Depression: a meta-analysis over a period of 49 years. Psychiatry Research, 30 <https://doi.org/10.1016/j.psychres.2010.12.007>
