# Supplementary material for: Effectiveness of cognitive behavioural therapy-based interventions for maternal perinatal depression: a systematic review and meta-analysis
Source: BMC Psychiatry. 2023 Mar 29;23:208. doi: 10.1186/s12888-023-04547-9 (PMC10052839; doi:10.1186/s12888-023-04547-9)
Supplement: Supplementary file 3 — Additional file 3. Electronic literature search strategies. [file 12888_2023_4547_MOESM3_ESM.docx]

S3. Electronic literature search strategies

| **Medline via Pubmed 1996-present - Provider: United States National Library of Medicine (NLM) – Date: 2021.11.25** |
| --- |
| ((((((((((((((((((((((((((((((((((((((((((postpartum[Title/Abstract]) OR post-partum[Title/Abstract]) OR antepartum[Title/Abstract]) OR ante-partum[Title/Abstract]) OR partum[Title/Abstract]) OR prepartum[Title/Abstract]) OR pre-partum[Title/Abstract]) OR intrapartum[Title/Abstract]) OR intra-partum[Title/Abstract]) OR peripartum[Title/Abstract]) OR peri-partum[Title/Abstract]) OR postnatal[Title/Abstract]) OR post-natal[Title/Abstract]) OR perinatal[Title/Abstract]) OR peri-natal[Title/Abstract]) OR antenatal[Title/Abstract]) OR ante-natal[Title/Abstract]) OR prenatal[Title/Abstract]) OR pre-natal[Title/Abstract]) OR pregnant[Title/Abstract]) OR pregnancy[Title/Abstract]) OR pregnancies[Title/Abstract]) OR puerper*[Title/Abstract]) OR maternal[Title/Abstract]) OR trimester[Title/Abstract]) OR impregnated[Title/Abstract]) OR gravid*[Title/Abstract]) OR multigravid*[Title/Abstract]) OR primigravid*[Title/Abstract]) OR parity[Title/Abstract]) OR obstetric[Title/Abstract]) OR gestation[Title/Abstract]) OR "in utero"[Title/Abstract]) OR maternity[Title/Abstract]) OR partus[Title/Abstract]) OR obstetrical[Title/Abstract])) OR ((Pregnancy[MeSH Terms]) OR postpartum period[MeSH Terms]))) AND ((((((((((((((((((depression[Title/Abstract]) OR depressed[Title/Abstract]) OR depressive[Title/Abstract]) OR "low mood"[Title/Abstract]) OR mood[Title/Abstract]) OR distress[Title/Abstract]) OR wellbeing[Title/Abstract]) OR "well being"[Title/Abstract]) OR emotion[Title/Abstract]) OR emotional[Title/Abstract]) OR melanchol*[Title/Abstract]) OR affect[Title/Abstract]) OR affective[Title/Abstract]) OR dysphori*[Title/Abstract]) OR dysthymia[Title/Abstract]) OR alexithymia[Title/Abstract])) OR ((((((((((((affect[MeSH Terms]) OR mood disorders[MeSH Terms]) OR depression[MeSH Terms]) OR depressive disorder[MeSH Terms]) OR affective symptoms[MeSH Terms]) OR affective disorder[MeSH Terms]) OR depression, postpartum[MeSH Terms]) OR Prenatal Care/psychology[MeSH Terms]) OR Perinatal Care/psychology[MeSH Terms]) OR Postnatal Care/psychology[MeSH Terms]) OR Pregnancy Complications/psychology[MeSH Terms]) OR Pregnancy Complications/therapy[MeSH Terms]))) AND ((((((((((((((((cognitive[Title/Abstract]) OR behavior[Title/Abstract]) OR behavioral[Title/Abstract]) OR behaviour[Title/Abstract]) OR behavioural[Title/Abstract]) OR cognitive behavio*[Title/Abstract]) OR "behavioural activation"[Title/Abstract]) OR "behavioral activation"[Title/Abstract]) OR "problem solving"[Title/Abstract]) OR ccbt[Title/Abstract]) OR icbt[Title/Abstract]) OR "cognitive restructuring"[Title/Abstract]) OR "cognitive reframing"[Title/Abstract]) OR "activity scheduling"[Title/Abstract])) OR (((behavior therapy[MeSH Terms]) OR cognitive therapy[MeSH Terms]) OR behavior therapies, cognitive[MeSH Terms]))) AND ((((((((((((((((((((((((((((((((((((((therapy[Title/Abstract]) OR therapies[Title/Abstract]) OR psychotherapy[Title/Abstract]) OR intervention[Title/Abstract]) OR Management[Title/Abstract]) OR "program evaluation"[Title/Abstract]) OR program[Title/Abstract]) OR programs[Title/Abstract]) OR programme[Title/Abstract]) OR programmes[Title/Abstract]) OR group[Title/Abstract]) OR course[Title/Abstract]) OR online[Title/Abstract]) OR Internet[Title/Abstract]) OR web[Title/Abstract]) OR "web based"[Title/Abstract]) OR phone[Title/Abstract]) OR telephone[Title/Abstract]) OR skype[Title/Abstract]) OR e-therapy[Title/Abstract]) OR etherapy[Title/Abstract]) OR "computer assisted"[Title/Abstract]) OR "internet intervention"[Title/Abstract]) OR computer[Title/Abstract]) OR computerised[Title/Abstract]) OR computerized[Title/Abstract]) OR mobile[Title/Abstract]) OR tablet[Title/Abstract]) OR smartphone[Title/Abstract]) OR "internet administered"[Title/Abstract]) OR e-mental health[Title/Abstract]) OR m-mental health[Title/Abstract]) OR Ehealth[Title/Abstract]) OR e-health[Title/Abstract])) OR e-intervention[Title/Abstract])) OR (((Prenatal Care/methods[MeSH Terms]) OR Perinatal Care/methods[MeSH Terms]) OR Postnatal Care/methods[MeSH Terms]))) AND (((((((randomized controlled trial[Publication Type]) OR systematic review[Publication Type]) OR controlled clinical trial[Publication Type]) OR meta-analysis[Publication Type])) OR (((((((((((((((((((("randomized controlled trial"[Title/Abstract]) OR "randomized control trial"[Title/Abstract]) OR RCT[Title/Abstract]) OR controlled[Title/Abstract]) OR randomised[Title/Abstract]) OR randomized[Title/Abstract]) OR randomisation[Title/Abstract]) OR randomization[Title/Abstract]) OR "random assignment"[Title/Abstract]) OR "random allocation"[Title/Abstract]) OR random[Title/Abstract]) OR randomly[Title/Abstract]) OR control[Title/Abstract]) OR feasibility[Title/Abstract]) OR pilot[Title/Abstract]) OR "comparative study"[Title/Abstract]) OR "follow up"[Title/Abstract]) OR meta-analysis[Title/Abstract]) OR "meta analysis"[Title/Abstract]) OR Review[Title/Abstract])) OR ((((((allocation, random[MeSH Terms]) OR randomized controlled trial[MeSH Terms]) OR meta analysis[MeSH Terms]) OR randomized controlled trials as topic[MeSH Terms]) OR meta analysis as topic[MeSH Terms]) OR review, systematic[MeSH Terms])) |

| **ISI Web of Science Core Collection (1900-present). Including Conference Proceedings Citation Index- Science (CPCI-S) -1990-present**  **Conference Proceedings Citation Index- Social Science & Humanities (CPCI-SSH) -1990-Present via Clarivate Analytics- Date: 2021.11.25** |
| --- |
| TI=(postpartum OR post-partum OR antepartum OR ante-partum OR partum OR prepartum OR pre-partum OR intrapartum OR intra-partum OR peripartum OR peri-partum OR postnatal OR post-natal OR perinatal OR peri-natal OR antenatal OR ante-natal OR prenatal OR pre-natal OR pregnant OR pregnancy OR pregnancies OR puerper* OR maternal OR trimester OR impregnated OR gravid* OR multigravid* OR primigravid* OR parity OR obstetric OR gestation OR “in utero” OR maternity OR partus OR obstetrical) AND TI=(depression OR depressed OR depressive OR “low mood” OR mood OR distress OR wellbeing OR well-being OR emotion OR emotional OR melanchol* OR affect OR affective OR dysphori* OR dysthymia OR alexithymia) AND TI=(cognitive OR behaviour OR behavioural OR behaviour OR behavioural OR “cognitive behavio*” OR “behavioural activation” OR “behavioral activation” OR “problem solving” OR ccbt OR icbt OR “cognitive restructuring” OR “cognitive reframing” OR “activity scheduling”) AND TI=(therapy OR therapies OR psychotherapy OR intervention OR management OR “program evaluation” OR program OR programs OR programme OR programmes OR group OR course OR online OR internet OR web OR web-based OR phone OR telephone OR skype OR e-therapy OR etherapy OR “computer assisted” OR “internet intervention” OR computer OR computerised OR computerized OR mobile OR tablet OR smartphone OR “internet administered” OR “e-mental health” OR “m-mental health” OR Ehealth OR e-health OR e-intervention) AND TI=(“randomized controlled trial” OR “randomized control trial” OR RCT OR controlled OR randomised OR randomized OR randomisation OR randomization OR “random assignment” OR “random allocation” OR random OR randomly OR control OR feasibility OR pilot OR “comparative study” OR “follow up” OR meta-analysis OR “meta analysis” OR review) OR AB=(postpartum OR post-partum OR antepartum OR ante-partum OR partum OR prepartum OR pre-partum OR intrapartum OR intra-partum OR peripartum OR peri-partum OR postnatal OR post-natal OR perinatal OR peri-natal OR antenatal OR ante-natal OR prenatal OR pre-natal OR pregnant OR pregnancy OR pregnancies OR puerper* OR maternal OR trimester OR impregnated OR gravid* OR multigravid* OR primigravid* OR parity OR obstetric OR gestation OR “in utero” OR maternity OR partus OR obstetrical) AND AB=(depression OR depressed OR depressive OR “low mood” OR mood OR distress OR wellbeing OR well-being OR emotion OR emotional OR melanchol* OR affect OR affective OR dysphori* OR dysthymia OR alexithymia) AND AB=(cognitive OR behaviour OR behavioural OR behaviour OR behavioural OR “cognitive behavio*” OR “behavioural activation” OR “behavioral activation” OR “problem solving” OR ccbt OR icbt OR “cognitive restructuring” OR “cognitive reframing” OR “activity scheduling”) AND AB=(therapy OR therapies OR psychotherapy OR intervention OR management OR “program evaluation” OR program OR programs OR programme OR programmes OR group OR course OR online OR internet OR web OR web-based OR phone OR telephone OR skype OR e-therapy OR etherapy OR “computer assisted” OR “internet intervention” OR computer OR computerised OR computerized OR mobile OR tablet OR smartphone OR “internet administered” OR “e-mental health” OR “m-mental health” OR Ehealth OR e-health OR e-intervention) AND AB=(“randomized controlled trial” OR “randomized control trial” OR RCT OR controlled OR randomised OR randomized OR randomisation OR randomization OR “random assignment” OR “random allocation” OR random OR randomly OR control OR feasibility OR pilot OR “comparative study” OR “follow up” OR meta-analysis OR “meta analysis” OR review)  *Indexes=SCI-EXPANDED, SSCI, A&HCI, CPCI-S, CPCI-SSH, ESCI Timespan=All years* |

| **Cumulative Index to Nursing and Allied Health Literature (CINAHL) via EBSCO host - 1981-Present - Date: 2021.11.25** |
| --- |
| TI ( postpartum OR post-partum OR antepartum OR ante-partum OR partum OR prepartum OR pre-partum OR intrapartum OR intra-partum OR peripartum OR peri-partum OR postnatal OR post-natal OR perinatal OR peri-natal OR antenatal OR ante-natal OR prenatal OR pre-natal OR pregnant OR pregnancy OR pregnancies OR puerper* OR maternal OR trimester OR impregnated OR gravid* OR multigravid* OR primigravid* OR parity OR obstetric OR gestation OR “in utero” OR maternity OR partus OR obstetrical OR MH "Pregnancy" ) AND TI ( depression OR depressed OR depressive OR “low mood” OR mood OR distress OR wellbeing OR “well being” OR emotion OR emotional OR melanchol* OR affect OR affective OR dysphori* OR dysthymia OR alexithymia OR MH "Depression, Postpartum" OR MH "Depression" ) AND TI ( cognitive OR behaviour OR behavioural OR behaviour OR behavioural OR cognitive W2 behavio* OR “behavioural activation” OR “behavioral activation” OR “problem solving” OR ccbt OR icbt OR “cognitive restructuring” OR “cognitive reframing” OR “activity scheduling” OR MH "Cognitive Therapy" OR MH "Behavior Therapy" ) AND TI ( therapy OR therapies OR psychotherapy OR intervention OR management OR “program evaluation” OR program OR programs OR programme OR programmes OR group OR course OR online OR internet OR web OR “web based” OR phone OR telephone OR skype OR e-therapy OR etherapy OR “computer assisted” OR “internet intervention” OR computer OR computerised OR computerized OR mobile OR tablet OR smartphone OR “internet administered” OR “e-mental health” OR “m-mental health” OR Ehealth OR “e-health” OR “e-intervention” ) AND TI ( “randomized controlled trial” OR “randomized control trial” OR RCT OR controlled OR randomised OR randomized OR randomisation OR randomization OR “random assignment” OR “random allocation” OR random OR randomly OR control OR feasibility OR pilot OR “comparative study” OR “follow up” OR meta-analysis OR “meta analysis” OR review OR MH "Randomized Controlled Trials" OR MH "Pilot Studies" OR MH "Meta Analysis" OR MH "Systematic Review" ) OR AB ( postpartum OR post-partum OR antepartum OR ante-partum OR partum OR prepartum OR pre-partum OR intrapartum OR intra-partum OR peripartum OR peri-partum OR postnatal OR post-natal OR perinatal OR peri-natal OR antenatal OR ante-natal OR prenatal OR pre-natal OR pregnant OR pregnancy OR pregnancies OR puerper* OR maternal OR trimester OR impregnated OR gravid* OR multigravid* OR primigravid* OR parity OR obstetric OR gestation OR “in utero” OR maternity OR partus OR obstetrical OR MH "Pregnancy" ) AND AB ( depression OR depressed OR depressive OR “low mood” OR mood OR distress OR wellbeing OR “well being” OR emotion OR emotional OR melanchol* OR affect OR affective OR dysphori* OR dysthymia OR alexithymia OR MH "Depression, Postpartum" OR MH "Depression" ) AND AB ( cognitive OR behaviour OR behavioural OR behaviour OR behavioural OR cognitive W2 behavio* OR “behavioural activation” OR “behavioral activation” OR “problem solving” OR ccbt OR icbt OR “cognitive restructuring” OR “cognitive reframing” OR “activity scheduling” OR MH "Cognitive Therapy" OR MH "Behavior Therapy" ) AND AB ( therapy OR therapies OR psychotherapy OR intervention OR management OR “program evaluation” OR program OR programs OR programme OR programmes OR group OR course OR online OR internet OR web OR “web based” OR phone OR telephone OR skype OR e-therapy OR etherapy OR “computer assisted” OR “internet intervention” OR computer OR computerised OR computerized OR mobile OR tablet OR smartphone OR “internet administered” OR “e-mental health” OR “m-mental health” OR Ehealth OR “e-health” OR “e-intervention” ) AND AB ( “randomized controlled trial” OR “randomized control trial” OR RCT OR controlled OR randomised OR randomized OR randomisation OR randomization OR “random assignment” OR “random allocation” OR random OR randomly OR control OR feasibility OR pilot OR “comparative study” OR “follow up” OR meta-analysis OR “meta analysis” OR review OR MH "Randomized Controlled Trials" OR MH "Pilot Studies" OR MH "Meta Analysis" OR MH "Systematic Review" ) |

| **Cochrane Central Register of Controlled Trials (CENTRAL) 1996-present – Date: 2021.11.25** | |
| --- | --- |
| **#** | **Searches** |
| #1 | (postpartum OR post-partum OR antepartum OR ante-partum OR partum OR prepartum OR pre-partum OR intrapartum OR intra-partum OR peripartum OR peri-partum OR postnatal OR post-natal OR perinatal OR peri-natal OR antenatal OR ante-natal OR prenatal OR pre-natal OR pregnant OR pregnancy OR pregnancies OR puerper* OR maternal OR trimester OR impregnated OR gravid* OR multigravid* OR primigravid* OR parity OR obstetric OR gestation OR “in utero” OR maternity OR partus OR obstetrical):ti,ab,kw |
| #2 | (depression OR depressed OR depressive OR "low mood" OR mood OR distress OR wellbeing OR "well-being" OR emotion OR emotional OR melanchol* OR affect OR affective OR dysphori* OR dysthymia OR alexithymia):ti,ab,kw |
| #3 | (cognitive OR behaviour OR behavioural OR behaviour OR behavioural OR cognitive NEAR/2 behavio* OR “behavioural activation” OR “behavioral activation” OR “problem solving” OR ccbt OR icbt OR “cognitive restructuring” OR “cognitive reframing” OR “activity scheduling”):ti,ab,kw |
| #4 | (therapy OR therapies OR psychotherapy OR intervention OR management OR “program evaluation” OR program OR programs OR programme OR programmes OR group OR course OR online OR internet OR web OR “web based” OR phone OR telephone OR skype OR e-therapy OR etherapy OR “computer assisted” OR “internet intervention” OR computer OR computerised OR computerized OR mobile OR tablet OR smartphone OR “internet administered” OR “e-mental health” OR “m-mental health” OR Ehealth OR “e-health” OR “e-intervention”):ti,ab,kw |
| #5 | (“randomized controlled trial” OR “randomized control trial” OR RCT OR controlled OR randomised OR randomized OR randomisation OR randomization OR “random assignment” OR “random allocation” OR random OR randomly OR control OR feasibility OR pilot OR “comparative study” OR “follow up” OR meta-analysis OR “meta analysis” OR review):ti,ab,kw |
| #6 | #1 AND #2 AND #3 AND #4 AND #5 |

| **PROSPERO: International prospective register of systematic reviews – 2011-present Date: 2021.11.25** |
| --- |
| postpartum OR post-partum OR antepartum OR ante-partum OR partum OR prepartum OR pre-partum OR intrapartum OR intra-partum OR peripartum OR peri-partum OR postnatal OR post-natal OR perinatal OR peri-natal OR antenatal OR ante-natal OR prenatal OR pre-natal OR pregnant OR pregnancy OR pregnancies OR puerper* OR maternal OR trimester OR impregnated OR gravid* OR multigravid* OR primigravid* OR parity OR obstetric OR gestation OR in utero OR maternity OR partus OR obstetrical AND depression OR depressed OR depressive OR low mood OR mood OR distress OR wellbeing OR well-being OR emotion OR emotional OR melanchol* OR affect OR affective OR dysphori* OR dysthymia OR alexithymia AND cognitive OR behaviour OR behavioural OR behaviour OR behavioural OR cognitive behavio* OR behavioural activation OR behavioral activation OR problem solving OR ccbt OR icbt OR cognitive restructuring OR cognitive reframing OR activity scheduling AND therapy OR therapies OR psychotherapy OR intervention OR management OR program evaluation OR program OR programs OR programme OR programmes OR group OR course OR online OR internet OR web OR web-based OR phone OR telephone OR skype OR e-therapy OR etherapy OR computer-assisted OR internet intervention OR computer OR computerised OR computerized OR mobile OR tablet OR smartphone OR internet-administered OR e-mental health OR m-mental health OR Ehealth OR e-health OR e-intervention AND randomized controlled trial OR randomized control trial OR RCT OR controlled OR randomised OR randomized OR randomisation OR randomization OR random assignment OR random allocation OR random OR randomly OR control OR feasibility OR pilot OR comparative study OR follow up:HA |

| **Excerpta Medica database (Embase) via OVID 1947-present – Date: 2021.11.25** | | |
| --- | --- | --- |
| **#** | **Searches** | **Results** |
| #1 | (postpartum OR post-partum OR antepartum OR ante-partum OR partum OR prepartum OR pre-partum OR intrapartum OR intra-partum OR peripartum OR peri-partum OR postnatal OR post-natal OR perinatal OR peri-natal OR antenatal OR ante-natal OR prenatal OR pre-natal OR pregnant OR pregnancy OR pregnancies OR puerper* OR maternal OR trimester OR impregnated OR gravid* OR multigravid* OR primigravid* OR parity OR obstetric OR gestation OR in utero OR maternity OR partus OR obstetrical).ti,ab. |  |
| #2 | exp pregnancy/ |  |
| #3 | postpartum period.mp. |  |
| #4 | puerperium/ |  |
| #5 | #1 OR #2 OR #3 OR #4 |  |
| #6 | (depression OR depressed OR depressive OR low mood OR mood OR distress OR wellbeing OR well-being OR emotion OR emotional OR melanchol* OR affect OR affective OR dysphori* OR dysthymia OR alexithymia).ti,ab. |  |
| #7 | exp mood disorder/ |  |
| #8 | exp depression/ |  |
| #9 | exp postnatal depression/ |  |
| #10 | #6 OR #7 OR #8 OR #9 |  |
| #11 | (cognitive OR behaviour OR behavioural OR behaviour OR behavioural OR cognitive ADJ2 behavio* OR behavioural activation OR behavioral activation OR problem solving OR ccbt OR icbt OR cognitive restructuring OR cognitive reframing OR activity scheduling).ti,ab. |  |
| #12 | exp cognitive behavioral therapy/ |  |
| #13 | #12 OR #13 |  |
| #16 | (therapy OR therapies OR psychotherapy OR intervention OR management OR program evaluation OR program OR programs OR programme OR programmes OR group OR course OR online OR internet OR web OR web based OR phone OR telephone OR skype OR e-therapy OR etherapy OR computer assisted OR internet intervention OR computer OR computerised OR computerized OR mobile OR tablet OR smartphone OR internet administered OR e-mental health OR m-mental health OR Ehealth OR e-health OR e-intervention).ti,ab. |  |
| #17 | (randomized controlled trial OR randomized control trial OR RCT OR controlled OR randomised OR randomized OR randomisation OR randomization OR random assignment OR random allocation OR random OR randomly OR control OR feasibility OR pilot OR comparative study OR follow up OR meta-analysis OR meta analysis OR review).ti,ab. |  |
| #18 | exp randomization/ |  |
| #19 | controlled clinical trial/ |  |
| #20 | exp "systematic review"/ |  |
| #22 | #17 OR #18 OR #19 OR #20 OR #21 |  |
| #23 | #4 AND #11 AND #15 AND #16 AND #22 |  |

| **Applied Social Sciences Index & Abstracts (ASSIA) via ProQuest 1987-present – Date: 2021.11.25** |
| --- |
| AB("postpartum" OR "post-partum" OR "antepartum" OR "ante-partum" OR "partum" OR "prepartum" OR "pre-partum" OR "intrapartum" OR "intra-partum" OR "peripartum" OR "peri-partum" OR "postnatal" OR "post-natal" OR "perinatal" OR "peri-natal" OR "antenatal" OR "ante-natal" OR "prenatal" OR "pre-natal" OR "pregnant" OR "pregnancy" OR "pregnancies" OR "puerper*" OR "maternal" OR "trimester" OR "impregnated" OR "gravid*" OR "multigravid*" OR "primigravid*" OR "parity" OR "obstetric" OR "gestation" OR “in utero” OR "maternity" OR "partus" OR "obstetrical")OR TI("postpartum" OR "post-partum" OR"antepartum" OR "ante-partum" OR "partum" OR "prepartum" OR "pre-partum" OR "intrapartum" OR "intra-partum" OR "peripartum" OR "peri-partum" OR "postnatal" OR "post-natal" OR "perinatal" OR "peri-natal" OR "antenatal" OR "ante-natal" OR "prenatal" OR "pre-natal" OR "pregnant" OR "pregnancy" OR "pregnancies" OR "puerper*" OR "maternal" OR "trimester" OR "impregnated" OR "gravid*" OR "multigravid*" OR "primigravid*" OR "parity" OR "obstetric" OR "gestation" OR “in utero” OR "maternity" OR "partus" OR "obstetrical") OR MAINSUBJECT.EXACT.EXPLODE("Pregnancy") OR MAINSUBJECT.EXACT.EXPLODE("Postpartum women")  AND AB("depression” OR “depressed” OR “depressive” OR “low mood” OR “mood” OR “distress” OR “wellbeing” OR “well-being” OR “emotion” OR “emotional” OR “melanchol*” OR “affect” OR “affective” OR “dysphori*” OR “dysthymia” OR “alexithymia”) OR TI("depression” OR “depressed” OR “depressive” OR “low mood” OR “mood” OR “distress” OR “wellbeing” OR “well-being” OR “emotion” OR “emotional” OR “melanchol*” OR “affect” OR “affective” OR “dysphori*” OR “dysthymia” OR “alexithymia”) AND AB(“cognitive” OR “behaviour” OR “behavioural” OR “behaviour” OR “behavioural” OR “cognitive behavio*” OR “behavioural activation” OR “behavioral activation” OR “problem solving” OR “ccbt” OR “icbt” OR “cognitive restructuring” OR “cognitive reframing” OR “activity scheduling”) OR TI(“cognitive” OR “behaviour” OR “behavioural” OR “behaviour” OR “behavioural” OR “cognitive behavio*” OR “behavioural activation” OR “behavioral activation” OR “problem solving” OR “ccbt” OR “icbt” OR “cognitive restructuring” OR “cognitive reframing” OR “activity scheduling”) OR MAINSUBJECT.EXACT.EXPLODE("Cognitive behaviour therapy") AND AB(“therapy” OR “therapies” OR “psychotherapy” OR “intervention” OR “management” OR “program evaluation” OR “program” OR “programs” OR “programme” OR “programmes” OR “group” OR “course” OR “online” OR “internet” OR “web” OR “web-based” OR “phone” OR “telephone” OR “skype” OR “e-therapy” OR “etherapy” OR “computer-assisted” OR “internet intervention” OR “computer” OR “computerised” OR “computerized” OR “mobile” OR “tablet” OR “smartphone” OR “internet-administered” OR “e-mental health” OR “m-mental health” OR “Ehealth” OR “e-health” OR “e-intervention”) OR TI(“therapy” OR “therapies” OR “psychotherapy” OR “intervention” OR “management” OR “program evaluation” OR “program” OR “programs” OR “programme” OR “programmes” OR “group” OR “course” OR “online” OR “internet” OR “web” OR “web-based” OR “phone” OR “telephone” OR “skype” OR “e-therapy” OR “etherapy” OR “computer-assisted” OR “internet intervention” OR “computer” OR “computerised” OR “computerized” OR “mobile” OR “tablet” OR “smartphone” OR “internet-administered” OR “e-mental health” OR “m-mental health” OR “Ehealth” OR “e-health” OR “e-intervention”) AND AB(“randomized controlled trial” OR “randomized control trial” OR “RCT” OR “controlled” OR “randomised” OR “randomized” OR “randomisation” OR “randomization” OR “random assignment” OR “random allocation” OR “random” OR “randomly” OR “control” OR “feasibility” OR “pilot” OR “comparative study” OR “follow up” OR “meta-analysis” OR “metaanalysis” OR “review”) OR TI(“randomized controlled trial” OR “randomized control trial” OR “RCT” OR “controlled” OR “randomised” OR “randomized” OR “randomisation” OR “randomization” OR “random assignment” OR “random allocation” OR “random” OR “randomly” OR “control” OR “feasibility” OR “pilot” OR “comparative study” OR “follow up” OR “meta-analysis” OR “metaanalysis” OR “review”) OR MAINSUBJECT.EXACT("Randomized controlled trials") OR MAINSUBJECT.EXACT.EXPLODE("Pilot studies") OR MAINSUBJECT.EXACT.EXPLODE("Followup") OR MAINSUBJECT.EXACT.EXPLODE("Meta-analysis") |

| **Psychinfo via EBSCO host -1967-present Date: 2021.11.25** | |
| --- | --- |
| **#** | **Searches** |
| 1 | (postpartum OR post-partum OR antepartum OR ante-partum OR partum OR prepartum OR pre-partum OR intrapartum OR intra-partum OR peripartum OR peri-partum OR postnatal OR post-natal OR perinatal OR peri-natal OR antenatal OR ante-natal OR prenatal OR pre-natal OR pregnant OR pregnancy OR pregnancies OR puerper* OR maternal OR trimester OR impregnated OR gravid* OR multigravid* OR primigravid* OR parity OR obstetric OR gestation OR in utero OR maternity OR partus OR obstetrical).ti,ab. |
| 2 | (depression OR depressed OR depressive OR low mood OR mood OR distress OR wellbeing OR well-being OR emotion OR emotional OR melanchol* OR affect OR affective OR dysphori* OR dysthymia OR alexithymia).ti,ab. |
| 3 | (cognitive OR behaviour OR behavioural OR behaviour OR behavioural OR cognitive behavio* OR behavioural activation OR behavioral activation OR problem solving OR ccbt OR icbt OR cognitive restructuring OR cognitive reframing OR activity scheduling).ti,ab. |
| 4 | (therapy OR therapies OR psychotherapy OR intervention OR management OR program evaluation OR program OR programs OR programme OR programmes OR group OR course OR online OR internet OR web OR web-based OR phone OR telephone OR skype OR e-therapy OR etherapy OR computer-assisted OR internet intervention OR computer OR computerised OR computerized OR mobile OR tablet OR smartphone OR internet-administered OR e-mental health OR m-mental health OR Ehealth OR e-health OR e-intervention).ti,ab. |
| 5 | (randomized controlled trial OR randomized control trial OR RCT OR controlled OR randomised OR randomized OR randomisation OR randomization OR random assignment OR random allocation OR random OR randomly OR control OR feasibility OR pilot OR comparative study OR follow up OR meta-analysis OR meta analysis OR review).ti,ab. |
| 6 | 1 AND 2 AND 3 AND 4 AND 5 |

| **Scopus 1966-present – Date: 2021.11.25** |
| --- |
| TITLE-ABS (postpartum OR post-partum OR antepartum OR ante-partum OR partum OR prepartum OR pre-partum OR intrapartum OR intra-partum OR peripartum OR peri-partum OR postnatal OR post-natal OR perinatal OR peri-natal OR antenatal OR ante-natal OR prenatal OR pre-natal OR pregnant OR pregnancy OR pregnancies OR puerper* OR maternal OR trimester OR impregnated OR gravid* OR multigravid* OR primigravid* OR parity OR obstetric OR gestation OR "in utero" OR maternity OR partus OR obstetrical) AND TITLE-ABS (depression OR depressed OR depressive OR "low mood" OR mood OR distress OR wellbeing OR "well-being" OR emotion OR emotional OR melanchol* OR affect OR affective OR dysphori* OR dysthymia OR alexithymia) AND TITLE-ABS (cognitive OR behaviour OR behavioural OR behaviour OR behavioural OR cognitive W/2 behavio* OR "behavioural activation" OR "behavioral activation" OR "problem solving" OR ccbt OR icbt OR "cognitive restructuring" OR "cognitive reframing" OR "activity scheduling") AND TITLE-ABS (therapy OR therapies OR psychotherapy OR intervention OR management OR "program evaluation" OR program OR programs OR programme OR programmes OR group OR course OR online OR internet OR web OR "web-based" OR phone OR telephone OR skype OR "e-therapy" OR etherapy OR "computer assisted" OR "internet intervention" OR computer OR computerised OR computerized OR mobile OR tablet OR smartphone OR "internet administered" OR "e-mental health" OR "m-mental health" OR Ehealth OR "e-health" OR "e-intervention") AND TITLE-ABS ("randomized controlled trial" OR "randomized control trial" OR RCT OR controlled OR randomised OR randomized OR randomisation OR randomization OR "random assignment" OR "random allocation" OR random OR randomly OR control OR feasibility OR pilot OR "comparative study" OR "follow up" OR meta-analysis OR "meta analysis" OR review) |

| **Svemed+ via Karolinska Institutet - Date: 2020.01.17** (As of January 2020, Karolinska Institutet University Library no longer updates SveMed + with new material)  **Search** |
| --- |
| **English:**  (postpartum OR post-partum OR antepartum OR ante-partum OR partum OR prepartum OR pre-partum OR intrapartum OR intra-partum OR peripartum OR peri-partum OR postnatal OR post-natal OR perinatal OR peri-natal OR antenatal OR ante-natal OR prenatal OR pre-natal OR pregnant OR pregnancy OR pregnancies OR puerper* OR maternal OR trimester OR impregnated OR gravid* OR multigravid* OR primigravid* OR parity OR obstetric OR gestation OR "in utero" OR maternity OR partus OR obstetrical OR exp: “pregnancy” OR exp: postpartum period”) AND (depression OR depressed OR depressive OR "low mood" OR mood OR distress OR wellbeing OR "well-being" OR emotion OR emotional OR melanchol* OR affect OR affective OR dysphori* OR dysthymia OR alexithymia OR exp: “affect” OR exp: “mood disorders” OR exp: “depression” OR exp: “depressive disorder” OR exp: “affective symptoms” OR exp: “affective disorder” OR exp: “depression, postpartum” OR exp: “prenatal Care/psychology” OR exp: “perinatal Care/psychology” OR exp: “postnatal Care/psychology” OR exp: “pregnancy Complications/psychology” OR exp: “pregnancy Complications/therapy”) AND (cognitive OR behaviour OR behavioural OR behaviour OR behavioural OR cognitive behavio* OR "behavioural activation" OR "behavioral activation" OR "problem solving" OR ccbt OR icbt OR "cognitive restructuring" OR "cognitive reframing" OR "activity scheduling" OR exp: “behavior therapy” OR exp: “cognitive therapy” OR exp: “behavior therapies, cognitive”)  **Swedish:**  förlossningsdepression OR förlossning OR födande OR barnafödande  OR amningsdepression OR gravid OR multigravid OR primigravid OR  graviditet OR graviditeter OR graviditetsdepression OR havandeskap OR  havande OR förstföderskor OR mångföderskor OR mödrahälsa OR  graviditetstrimestrar OR trimester OR intrapartal OR prenatal OR  postpartum OR gestationsålder OR obstetrisk OR befruktan OR befruktad  OR befruktning OR exp:"graviditet" AND [depression OR depressive OR  deprimerad OR affektiva sjukdomar OR välmående OR känsla OR  känslomässig OR psykisk ohälsa OR melankoli OR melankolisk OR exp: "depression" OR exp: "depressiv sjukdom" OR exp: "emotionella  störningar" OR exp:"prenatal vård/psykologi" OR exp: "postnatal vård/psykologi" OR exp:"graviditetskomplikationer/terapi"  OR exp:"graviditetskomplikationer/psykologi"](https://svemedplus.kib.ki.se/Default.aspx?query=depression%20OR%20depressive%20OR%20deprimerad%20OR%20affektiva%20sjukdomar%20OR%20v%C3%A4lm%C3%A5ende%20OR%20k%C3%A4nsla%20OR%20k%C3%A4nslom%C3%A4ssig%20OR%20psykisk%20oh%C3%A4lsa%20OR%20melankoli%20OR%20melankolisk%20OR%20exp:%22depression%22%20OR%20exp:%20%22depressiv%20sjukdom%22%20OR%20exp:%20%22emotionella%20st%C3%B6rningar%22%20OR%20exp:%22prenatal%20v%C3%A5rd/psykologi%22%20OR%20exp:%22postnatal%20v%C3%A5rd/psykologi%22%20OR%20exp:%22graviditetskomplikationer/terapi%22%20OR%20exp:%22graviditetskomplikationer/psykologi%22) AND [kognitiv OR beteende  OR beteenden OR "kognitiv beteende terapi" OR "beteendeaktivering"  OR "problemlösning" OR KBT OR "aktivitetsplanering" OR exp:"kognitiv terapi"  OR exp:"beteende terapi, kognition"](https://svemedplus.kib.ki.se/Default.aspx?query=kognitiv%20OR%20beteende%20OR%20beteenden%20OR%20%22kognitiv%20beteende%20terapi%22%20OR%20%22beteendeaktivering%22%20OR%20%22probleml%C3%B6sning%22%20OR%20KBT%20OR%20%22aktivitetsplanering%22%20OR%20exp:%22kognitiv%20terapi%22%20OR%20exp:%22beteende%20terapi,%20kognition%22) |
