# Supplementary material for: Effectiveness of cognitive behavioural therapy-based interventions for maternal perinatal depression: a systematic review and meta-analysis
Source: BMC Psychiatry. 2023 Mar 29;23:208. doi: 10.1186/s12888-023-04547-9 (PMC10052839; doi:10.1186/s12888-023-04547-9)
Supplement: Supplementary file 5 — Additional file 5. References to papers in languages other than English or Swedish. [file 12888_2023_4547_MOESM5_ESM.docx]

**S5.** **References to papers in languages other than English or Swedish**

Chabrol, H., Teissedre, F., Saint-Jean, M., Teisseyre, N., Sistac, C., Michaud, C., & Roge, B. (2002). Detection, prevention and treatment of postpartum depression: A controlled study of 859 patients. *L’Encephale, 28*(1), 65–70. https://doi.org/ENC-02-2002-28-1-0013-7006-101019-ART8

Cheng, H. Y., Huang, T. Y., Chien, L. Y., Cheng, Y. F., & Chen, F. J. (2016). The effects of a mobile application social support program on postpartum perceived stress and depression. *Journal of Nursing, 63*(6), 52–60. https://doi.org/10.6224/JN.63.6.52

Golzar, A. A. K., Golizadeh, Z., Sohrabi, A., Kiani, M. A., Allizadeh, N. S., & Asgarnajad, S. (2013). Effectiveness of cognitive - behavioral therapy in the treatment of postpartum depression and three dynamic conflicts of dependency, anger and motherhood in postpartum depressed mothers. *Iranian Journal of Obstetrics, Gynaecology and Infertility, 16*(52), 8–17. https://doi.org/[ijogi.mums.ac.ir/article_895.html](https://ijogi.mums.ac.ir/article_895.html)

Hou, Y., Hu, P., Zhang, Y., Lu, Q., Wang, D., Yin, L.,… Zou, X. (2012). Combined cognitive behavior therapy with systematic family therapy in patients with mild to moderate postpartum depression. *Chinese Mental Health Journal, 26*(10), 741–747. https://doi.org/10.1590/1516-4446-2013-1170

Karamoozian, M., & Askarizadeh, G. (2013). Effectiveness of cognitive-behavioral stress management intervention on anxiety and depression during pregnancy. *Journal of Kerman University of Medical Sciences, 20*(6), 606–621. <http://jkmu.kmu.ac.ir/article_16361_27caa7ae2cfd87eb2933efaa42ffb700.pdf>

Kordi, M., Nasiri, S., Modares Gharavi, M., & Ebrahimzadeh, S. (2012). The effect of problem solving skills training on severity of depression symptoms in postpartum period. *Journal of Fundamentals of Mental Health, 14*(55). https://doi.org/jfmh.mums.ac.ir/article_940.html

Parsa, P., Ahangpour, P., Fatemeh, S., Soltanian, A., & Rahimi, A. (2016). The effect of counselling based on Problem Solving on postpartum depression among mothers attending to health centres of Hamadan city, 1395. *Journal of Urmia Nursing and Midwifey Faculty, 15*(95), 440–448.

https://doi.org/unmf.umsu.ac.ir/article-1-3286-en.html

Piacentini, D., Mirabella, F., Leveni, D., Primerano, G., Cattaneo, M., Biffi, G., … Gigantesco, A. (2011). Effectiveness of a manualized cognitive-behavioural intervention for postnatal depression. *Rivista Di Psichiatria, 46*(3), 187–194. https://doi.org/10.1708/889.9809

Safaralinezhad, A., Oveisi, S., Sarichlu, M. ebrahim, & Jourabchi, Z. (2018). Effect of cognitive-behavioral group therapy on gestational depression: A clinical trial. *Iranian Journal of Obstetrics, Gynecology and Infertility, 21*(2), 48-59. https://www.sid.ir/en/journal/ViewPaper.aspx?id=599066
