# Supplementary material for: Effectiveness of cognitive behavioural therapy-based interventions for maternal perinatal depression: a systematic review and meta-analysis
Source: BMC Psychiatry. 2023 Mar 29;23:208. doi: 10.1186/s12888-023-04547-9 (PMC10052839; doi:10.1186/s12888-023-04547-9)
Supplement: Supplementary file 6 — Additional file 6. References to dissertations. [file 12888_2023_4547_MOESM6_ESM.docx]

**S6. References to dissertations**

Adey, C. (2016). “*Enjoy your baby” Internet-based CBT for mothers with babies: a feasibility randomised control trial* [Doctoral dissertation, University of Glasgow]. http://theses.gla.ac.uk/http://theses.gla.ac.uk/7603/

Bagnall, K. M. (2014). *Long-term follow-up of Netmums HWD: A feasibility randomised controlled trial of telephone supported online behavioural activation for postnatal depression at 16 months post-randomisation* [Doctoral dissertation, University of Exeter]. https://ore.exeter.ac.uk/repository/bitstream/handle/10871/15289/BagnallK.pdf?sequence=1&isAllowed=y

Boath, E. (1999). *The cost and effectiveness of two alternative approaches to the treatment of postnatal depression* [Undergraduate dissertation, Keele University]. http://www.opengrey.eu/item/display/10068/614514

Chungu, H. M. (2017). *The effects of cognitive behavioural therapy on the improvement of the health of youth mothers with postpartum depression at the University teaching hospital of Lusaka, Zambia* [Masters dissertation, University of Zambia]. http://dspace.unza.zm/bitstream/handle/123456789/5654/FINAL corrected REPORT latest 2.pdf;jsessionid=09802D2D7D13AC7E5E620451E970C1A8?sequence=1

Dymond, M. M. (2006). *Predictors of postpartum depression and moderators of outcome following a preventive intervention* [Doctoral dissertation, Reading University]. http://www.opengrey.eu/item/display/10068/953334

Gumery Hays, M.-Ai. (2002). *Newborn observation and treatment of early maternal postpartum depression (clinical, theoretical and methodological issues)* [Doctoral dissertation, Université Lumiére]. http://www.opengrey.eu/item/display/10068/725557

Khan, S. (2012). *Developing a culturally adapted cognitive behavioural therapy based intervention for British Pakistani mothers with persistent postnatal depression* [Doctoral dissertation, University of Manschester]. https://search-proquest-com.ezproxy.its.uu.se/docview/1775430689/previewPDF/186D3ECF78414B62PQ/1?accountid=14715

Liebert, H. (2016). *Postpartum depression: Screening and treatment of low income women in a primary care setting* [Doctoral dissertation, The Wright Institute]. https://search-proquest-com.ezproxy.its.uu.se/docview/1943779732?pq-origsite=gscholar

Pugh, N. E. (2014). *A randomized controlled trial of a therapist assisted internet cognitive behaviour therapy program for women with postpartum depression* [Doctoral dissertation, University of Regina].https://ourspace.uregina.ca/bitstream/handle/10294/5461/Pugh_Nicole_200277350_PhD_PSYC_Fall2014.pdf?sequence=2&isAllowed=y

Sadigursky, A. (2018). *Move my mood: Development and evaluation of a mobile mental health self-help app using behavioural activation for women with postpartum depression*. [Doctoral dissertation, Alliant International University]. https://search-proquest-com.ezproxy.its.uu.se/docview/2050664240/abstract/66865E6E3E2545C9PQ/3?accountid=14715

Sockol, E. L. (2013). *The Development, treatment, and prevention of perinatal mood and anxiety disorders* [Doctoral dissertation, University of Pennsylvania]. http://repository.upenn.edu/edissertations/801

Swales, A. (2015). *Moderators and mediators of outcome in an internet-based behavioural activation trial for postnatal depression (Netmums)* [Doctoral dissertation, University of Exeter]. https://search.proquest.com/docview/1780278083/66865E6E3E2545C9PQ/2?accountid=14715

Teissedre, F. (2003). *Postpartum depressions: A comparative study of two screening programs, of prevention and of treatment of postpartum depression on 450 women* [Doctoral dissertation, Université de Toulouse-Le Mirail]. http://www.opengrey.eu/item/display/10068/739889
