# Supplementary material for: Effectiveness of cognitive behavioural therapy-based interventions for maternal perinatal depression: a systematic review and meta-analysis
Source: BMC Psychiatry. 2023 Mar 29;23:208. doi: 10.1186/s12888-023-04547-9 (PMC10052839; doi:10.1186/s12888-023-04547-9)
Supplement: Supplementary file 7 — Additional file 7. PICOS statement. [file 12888_2023_4547_MOESM7_ESM.docx]

**S7. PICOS statement**

| **PICOS** | **Eligibility Criteria** | ✓ |
| --- | --- | --- |
| Population | Adult women (aged ≥ 16 years) | 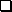 |
|  | Pregnant or post-partum (up to 12 months) | 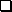 |
|  | Depressive disorder with peri-partum onset  For example:   - A diagnosis of major depression with perinatal onset specifier in accordance with the Diagnostic and Statistical Manual of Mental Disorders (DSM) IV or V, International Classification of Disease, Tenth Edition (ICD-10) (f53.0). - Reporting depression symptomatology with a peri-partum onset using a validated tool e.g., Edinburgh Postnatal Depression Scale (EPDS; Cox, 1987). | 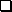 |
|  | *Exclusion criteria:* intervention for mood disorders other than depression (e.g. bipolar affective disorder). | 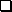  Does not contain |
|  | *Exclusion criteria:* interventions focussed on prevention of maternal psychopathology in at-risk, but not currently symptomatic mothers. | 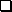  Does not contain |
| Intervention | Interventions explicitly targeting an improvement in peripartum depression e.g., not targeting mother-infant interaction.  *Note:* no exclusions are placed upon:   - Professional group supporting the intervention - Clinical setting of the intervention - Delivery mode (self-guided, individual or group) - Support methods (internet, face-to-face or telephone) | 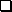 |
|  | Intervention explicitly states the use of cognitive behavioural therapy, behavioural activation and/or problem-solving.  For example:   - CBT will be defined as interventions in which the focus is modifying a client’s dysfunctional thoughts on current behaviour and future functioning (Cuijpers, van Straten, Andersson, & van Oppen, 2008). - BA will be defined as interventions targeting reductions in behavioural avoidance and increases in positively reinforcing activities, including interventions that focus on scheduling behaviours (Hopko, Lejuez, Ruggiero, & Eifert, 2003). - Problem-solving interventions will be defined as a psychological intervention including the following elements: definition of personal problems, generation of multiple solutions to each problem, selection of the best solution, developing a systematic plan for this solution, and evaluating whether the solution has resolved the problem (Cuijpers et al., 2008). | 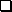 |
| Comparators | Trial contains a suitable control condition  For example:   - no-treatment control - wait-list control - treatment-as-usual - non-specific factors component control - specific factors component control - active comparator   *Note:* Only trial designs that allow for the isolation of the effects of CBT were included as it is important for active comparators to discriminate intervention effects (Evans 2010). For example, research comparing CBT alone versus medication alone is excluded as it would not be possible to isolate the effect of the CBT. | 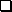 |
| Outcomes | Depression or peripartum depression is the primary outcome measure (self-report, clinician or proxy administered measure of depression) | 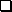 |
|  | Quality of depression measure used has internal consistency and test-retest reliability with a Cronbach’s alpha ≥0.70 or intra-class correlations | 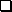 |
| Study design | Only randomized control trials | 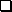 |
|  | Quality of randomization procedure based on Risk of Bias tool 2.0 (Higgins et al., 2018) and the CONSORT Statement 2010 (Schulz, Altman & Moher, 2010).  *Exclusion criteria:* explicitly stating the use of non-random sequence generation  *Exclusion criteria:* Designs in which explicitly state that the allocation sequence is not concealed (before participants are enrolled and assigned to interventions). | 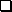  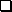 |
| Overall decision  Include Exclude Review  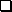 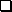 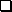 | | |
| Notes: | | |
