# Supplementary material for: Effectiveness of cognitive behavioural therapy-based interventions for maternal perinatal depression: a systematic review and meta-analysis
Source: BMC Psychiatry. 2023 Mar 29;23:208. doi: 10.1186/s12888-023-04547-9 (PMC10052839; doi:10.1186/s12888-023-04547-9)
Supplement: Supplementary file 8 — Additional file 8. Full paper PICOS screening. [file 12888_2023_4547_MOESM8_ESM.docx]

**S8. Full paper PICOS screening**

| **Study** | **Inclusion/Exclusion Criteria** | | | | | | | | | | | | | **Include/Exclude** |
| --- | --- | --- | --- | --- | --- | --- | --- | --- | --- | --- | --- | --- | --- | --- |
|  | **Adult women** | **Pregnant or postpartum** | **Peripartum onset** | **Exec: Other MD** | **Exclusion: Prevention** | **CBT, BA or PS** | **Targeting PPD** | **Control condition** | **Primary outcome** | **Outcome quality** | **RCT design** | **Exec:-Random sequence** | **Exec: Allocation concealment** |  |
| Alhusen et al., (2021) | I | I | I | I | I | I | I | I | I | I | I | I | I | I |
| Ammerman et al. (2013) | I | I | I | I | I | I | I | I | I | I | I | I | I | I |
| Anton and David (2015) | U | I | E | I | E | I | I | I | I | I | I | I | I | E |
| Appleby et al. (1997) | I | I | I | I | I | U | I | E | I | I | I | I | I | E |
| Araújo et al. (2016) | I | I | E | I | U | U | I | I | I | I | I | I | I | E |
| Asghari et al. (2016) | I | I | U | I | E | I | U | U | U | I | E | U | I | E |
| Austin et al. (2008) | I | I | E | I | I | I | I | E | I | I | I | I | I | E |
| Beattie et al. (2017) | I | E | E | I | U | E | E | I | I | I | I | I | I | E |
| Bernard et al. (2011) | I | I | E | I | I | I | I | I | I | I | I | I | I | E |
| Bhat et al. (2018) | I | I | I | I | I | I | I | E | I | I | E | E | E | E |
| Bittner et al. (2014) | I | I | E | I | E | I | I | I | U | I | I | I | I | E |
| Brugha et al. (2000) | I | I | E | I | E | I | I | I | I | I | I | I | I | E |
| Brugha et al. (2011) | I | I | E | U | E | I | I | I | I | I | I | I | I | E |
| Burns et al. (2013) | I | I | I | I | I | I | I | I | I | I | I | I | I | I |
| Carta et al. (2015) | I | I | I | I | E | I | I | E | I | I | E | E | E | E |
| Chabrol et al. (2002) | I | I | I | I | I | E | I | I | I | I | I | E | I | E |
| Chibanda et al. (2014) | I | I | I | U | I | E | I | E | I | I | I | I | I | E |
| Cho et al. (2008) | I | I | I | I | I | I | I | E | I | I | I | I | I | E |
| Cinciripini et al. (2010) | I | I | I | I | U | I | E | I | E | I | I | I | I | E |
| Cooper et al. (2003) | I | I | I | I | I | I | I | I | I | I | I | I | I | E |
| Dafei et al. (2021) | U | I | I | I | I | I | I | I | I | I | E | E | E | E |
| Danaher et al. (2013) | I | I | I | I | I | U | I | E | I | I | E | E | E | E |
| Dennis (2003) | I | I | U | I | U | E | I | I | I | I | I | I | I | E |
| Dimidjian et al. (2017) | I | I | I | I | I | I | I | I | I | I | I | I | I | I |
| Duffecy et al. (2019) | I | I | E | I | I | I | I | E | I | I | I | I | I | E |
| Fonseca et al. (2019) | I | I | E | I | E | E | E | I | I | I | I | I | I | E |
| Forsell et al. (2017) | I | I | I | I | I | I | I | I | I | I | I | I | I | I |
| Fuhr et al. (2019) | I | I | I | I | I | I | I | I | I | I | I | I | I | I |
| Futterman et al. (2010) | I | I | E | I | E | E | E | E | E | n/a | E | E | E | E |
| George et al. (2020) | U | I | I | I | E | I | I | I | I | I | I | I | I | E |
| Ginsburg et al. (2012) | E | I | E | I | E | I | I | U | I | I | I | I | I | E |
| Goodman et al. (2015) | I | I | E | I | I | E | I | I | I | I | I | I | I | E |
| Gureje et al. (2019) | I | I | I | I | I | I | I | E | I | I | I | I | I | E |
| Haga et al. (2019) | I | I | U | U | E | E | I | I | U | I | I | U | U | E |
| Heller et al. (2020) | I | I | I | E | I | I | E | I | I | I | I | I | I | E |
| Honey et al. (2002) | I | I | I | I | I | I | I | I | I | I | I | I | I | I |
| Hou et al. (2014) | I | I | I | I | I | I | I | I | I | I | I | E | I | E |
| Howell et al. (2012) | I | I | E | I | E | E | E | I | I | I | I | I | I | E |
| Howell et al. (2014) | I | I | E | I | E | E | I | I | I | I | I | I | I | E |
| Hughes et al. (2015) | I | I | I | I | I | I | I | I | I | I | I | I | I | I |
| Husain et al. (2017) | I | E | I | I | I | I | I | I | I | I | I | I | I | E |
| Husain et al. (2021) | I | I | E | I | I | I | I | I | I | I | I | I | I | E |
| Jannati et al. (2020)* | I | I | I | I | I | I | I | I | I | I | I | I | I | I |
| Jesse et al. (2015) | I | I | E | I | E | I | I | I | I | I | I | I | I | E |
| Jiang et al. (2014) | I | I | I | I | I | E | I | I | I | I | I | I | U | E |
| Kaaya et al. (2013) | I | I | E | I | E | I | E | I | I | U | I | I | I | E |
| Khamesh et al. (2019) | I | I | I | I | I | I | I | I | I | I | I | I | I | I |
| Kim et al. (2014) | I | I | I | I | I | I | I | E | I | I | E | E | E | E |
| Kingston et al. (2014) | U | I | I | I | I | I | E | I | U | I | E | E | E | E |
| Kozinsky et al. (2012) | I | I | E | I | E | U | I | I | I | U | I | I | U | E |
| Le et al. (2011) | I | I | E | I | E | I | I | I | I | I | I | U | I | E |
| Leung and Lam (2012) | I | I | E | I | E | E | E | I | E | I | I | U | U | E |
| Leung et al. (2013) | I | I | U | I | U | I | I | I | I | I | E | E | E | E |
| Leung et al. (2016) | I | I | I | I | I | I | I | E | I | I | I | I | I | E |
| Liu et al. (2021) | I | I | I | I | E | I | E | I | I | I | I | I | I | E |
| Loughnan et al. (2019) | I | I | E | I | I | I | E | I | I | I | I | I | I | E |
| Lowndes et al. (2019) | I | I | E | I | U | I | E | I | E | I | I | I | E | E |
| Lund et al., (2020) | I | I | I | I | I | I | I | I | I | I | I | I | I | I |
| Mao et al. (2012) | I | I | E | I | E | I | I | I | I | I | I | I | U | E |
| McKee et al. (2006) | I | I | I | I | I | I | I | I | I | I | I | I | I | I |
| Meager and Milgrom (1996) | I | I | I | I | I | I | I | I | I | I | I | I | I | I |
| Mehri et al. (2019) | E | E | U | U | I | I | I | I | I | I | I | U | U | E |
| Milgrom et al. (2011a) | I | I | U | I | U | E | U | I | U | I | I | I | I | E |
| Milgrom et al. (2011b) | I | I | I | I | I | I | I | I | I | I | I | I | I | I |
| Milgrom et al. (2015a) | I | I | I | I | I | I | I | I | I | I | I | I | I | I |
| Milgrom et al. (2015b) | I | I | I | I | I | I | I | I | I | I | I | I | I | I |
| Milgrom et al. (2016) | I | I | I | I | I | I | I | I | I | I | I | I | I | I |
| Milgrom et al. (2005) | I | I | I | I | I | I | I | I | I | I | I | I | I | I |
| Misri et al. (2004) | I | I | I | I | I | I | I | I | I | I | I | I | I | I |
| Morrell et al. (2009) | I | I | I | I | I | I | I | I | I | I | I | I | I | I |
| Muñoz et al. (2007) | I | I | E | I | E | I | E | I | I | I | I | I | I | E |
| Mureşan-Madar and Băban (2015) | I | I | I | I | I | I | I | I | I | I | I | E | I | E |
| Murray et al. (2003) | I | E | I | I | I | I | E | I | E | n/a | I | I | I | E |
| Nasiri et al. (2015) | I | I | I | I | I | I | E | I | E | n/a | I | I | I | E |
| Nasiri et al. (2018) | I | I | I | I | I | I | I | I | I | I | I | I | I | I |
| Ngai et al. (2015) | I | I | I | I | I | I | I | I | I | I | I | I | I | I |
| Ngai et al. (2020) | I | I | I | I | E | I | I | I | I | I | I | I | I | E |
| O’Mahen et al. (2013a) | I | I | I | I | I | I | I | I | I | I | I | I | I | I |
| O’Mahen et al. (2013b) | I | I | I | I | I | I | I | I | I | I | I | I | I | I |
| O’Mahen et al. (2014) | I | I | I | I | I | I | I | I | I | I | I | I | I | I |
| Ortiz et al. (2014) | I | I | E | I | E | E | E | I | I | I | I | I | I | E |
| Pinheiro et al. (2014) | I | U | I | U | I | I | I | E | I | I | I | I | I | E |
| Prendergast and Austin (2001) | I | I | I | I | I | I | U | E | U | I | I | I | I | E |
| Puckering et al. (2010) | U | I | I | I | I | E | U | I | U | I | I | U | U | E |
| Pugh et al. (2016) | I | I | I | I | I | I | I | I | I | I | I | I | I | I |
| Rahman et al. (2008) | I | I | I | I | I | I | I | U | E | I | I | U | U | E |
| Ramezani et al. (2017) | I | I | E | I | E | I | U | I | U | I | I | I | I | E |
| Rees et al. (1993) | I | I | E | I | E | E | I | I | I | E | I | U | U | E |
| Rojas et al. (2007) | I | I | I | I | I | I | I | I | I | I | I | I | I | I |
| Sanders and McFarland (2000) | I | E | E | I | I | I | E | U | E | E | I | I | I | E |
| Sandner et al. (2018) | U | U | E | E | E | E | E | I | E | E | I | I | I | E |
| Shamshiri Milani et al. (2015) | I | I | U | I | I | E | I | I | I | I | I | U | U | E |
| Sheeber et al. (2012) | I | E | E | I | U | I | I | I | I | I | I | I | I | E |
| Sikander et al. (2019) | I | I | I | I | I | I | I | I | I | I | I | I | I | I |
| Silverstein et al. (2011) | I | E | E | I | E | I | I | I | I | U | I | I | I | E |
| Silverstein et al. (2018) | U | U | U | I | U | I | U | I | E | E | I | I | I | E |
| Stein et al. (2018) | I | I | I | I | I | I | I | E | E | n/a | I | I | I | E |
| Surkan et al. (2012) | I | E | E | I | E | E | E | I | I | I | I | I | I | E |
| Tandon et al. (2014) | E | I | E | I | E | I | I | I | I | I | I | I | U | E |
| Tandon et al. (2011) | I | I | E | I | E | I | I | I | I | I | I | I | I | E |
| Tandon et al. (2018) | I | I | E | I | E | I | I | I | I | I | I | I | I | E |
| Trevillon et al. (2020) | I | I | I | I | I | I | I | I | I | I | I | I | I | I |
| Ugarriza and Schmidt (2006) | I | I | E | I | E | I | I | E | I | I | E | E | E | E |
| Ugarriza (2004) | I | I | I | I | I | I | I | U | I | I | I | E | U | E |
| Van Lieshout et al. (2017) | I | I | I | I | I | I | I | E | I | I | E | E | E | E |
| Van Lieshout et al. (2020) | I | I | I | I | I | I | I | E | I | I | E | E | E | E |
| Van Lieshout et al. (2021) | I | I | I | I | I | I | I | I | I | I | I | I | I | I |
| Van Ravesteyn et al. (2018) | I | I | I | I | E | U | I | E | I | I | I | I | I | E |
| Wiklund et al. (2010) | I | I | I | I | I | I | I | I | I | I | I | I | I | I |
| Wozney et al. (2017) | I | I | I | I | I | I | I | I | I | I | I | I | I | I |
| Yazdanimehr et al. (2016) | I | I | I | I | I | E | I | I | I | I | I | I | I | E |

*Note.:* Abbreviations: E: Exclude; I: Include; n/a; criteria does not apply (e.g. if an outcome measure was excluded the quality criteria does not apply); U; Unclear (information either missing or unclear form the paper, if no other exclusion criteria applied authors where contacted in order to clarify further and in cases where other exclusion criteria applied no further information was sought). *Excluded due to data irregularity
