# Supplementary material for: Effectiveness of cognitive behavioural therapy-based interventions for maternal perinatal depression: a systematic review and meta-analysis
Source: BMC Psychiatry. 2023 Mar 29;23:208. doi: 10.1186/s12888-023-04547-9 (PMC10052839; doi:10.1186/s12888-023-04547-9)
Supplement: Supplementary file 9 — Additional file 9. References for excluded studies. [file 12888_2023_4547_MOESM9_ESM.docx]

**S9. References for excluded studies**

Anton, R., & David, D. (2015). A randomized clinical trial of a new preventative rational emotive and behavioral therapeutical program of prepartum and postpartum emotional distress. *Journal of Evidence-Based Psychotherapies, 15*(1), 3-15. https://doi.org/[10.1007/s10156-012-0467-7](https://doi.org/10.1007/s10156-012-0467-7)

Appleby, L., Warner, R., Whitton, A., & Faragher, B. (1997). A controlled study of fluoxetine and cognitive-behavioural counselling in the treatment of postnatal depression. *British Medical Journal, 314*(7085), 932–936. https://doi.org/10.1136/bmj.314.7085.932

Araújo, W. S., Romero, W. G., Zandonade, E., & Amorim, M. H. (2016). Effects of relaxation on depression levels in women with high-risk pregnancies: A randomised clinical trial. *Revista Latino-Americana de Enfermagem*, *24*(0), e2806. https://doi.org/10.1590%2F1518-8345.1249.2806

Asghari, E., Faramarzi, M., & Mohammmadi, A. K. (2016). The effect of cognitive behavioural therapy on anxiety, depression and stress in women with preeclampsia. *Journal of Clinical and Diagnostic Research*, *10*(11). https://doi.org/10.7860%2FJCDR%2F2016%2F21245.8879

Austin, M. P., Frilingos, M., Lumley, J., Hadzi-Pavlovic, D., Roncolato, W., Acland, S., … & Parker, G. (2008). Brief antenatal cognitive behaviour therapy group intervention for the prevention of postnatal depression and anxiety: A randomised controlled trial. *Journal of Affective Disorders*, *105*(1-3), 35–44. https://doi.org/10.1016/j.jad.2007.04.001

Beattie, J., Hall, H., Biro, M. A., East, C., & Lau, R. (2017). Effects of mindfulness on maternal stress, depressive symptoms and awareness of present moment experience: A pilot randomised trial. *Midwifery*, *50*, 174–183. https://doi.org/10.1016/j.midw.2017.04.006

Bernard, R. S., Williams, S. E., Storfer-Isser, A., Rhine, W., Horwitz, S. M., Koopman, C., & Shaw, R. J. (2011). Brief cognitive-behavioral intervention for maternal depression and trauma in the neonatal intensive care unit: A pilot study. *Journal of Traumatic Stress*, *24*(2), 230–234. https://doi.org/10.1002/jts.20626

Bhat, A., Reed, S., Mao, J., Vredevoogd, M., Russo, J., Unger, J., … Unützer, J. (2018). Delivering perinatal depression care in a rural obstetric setting: A mixed methods study of feasibility, acceptability and effectiveness. *Journal of Psychosomatic Obstetrics and Gynecology*, *39*(4), 273–280. https://doi.org/10.1080/0167482X.2017.1367381

Bittner, A., Peukert, J., Zimmermann, C., Junge-Hoffmeister, J., Parker, L. S., Stöbel-Richter, Y., & Weidner, K. (2014). Early intervention in pregnant women with elevated anxiety and depressive symptoms: Efficacy of a cognitive-behavioral group program. *The Journal of Perinatal and Neonatal Nursing*, *28*(3), 185–195. https://doi.org/10.1097/JPN.0000000000000027

Brugha, T. S., Morrell, C. J., Slade, P., & Walters, S. J. (2011). Universal prevention of depression in women postnatally: Cluster randomized trial evidence in primary care. *Psychological Medicine*, *41*(4), 739–748. https://doi.org/10.1017/s0033291710001467

Brugha, T. S., Wheatley, S., Taub, N. A., Culverwell, A., Friedman, T., Kirwan, P., … Shapiro, D. A. (2000). Pragmatic randomized trial of antenatal intervention to prevent post-natal depression by reducing psychosocial risk factors. *Psychological Medicine*, *30*(6), 1273–1281. https://doi.org/10.1017/s0033291799002937

Carta, G., D'Alfonso, A., Parisse, V., Di Fonso, A., Casacchia, M., & Patacchiola, F. (2015). How does early cognitive behavioural therapy reduce postpartum depression? *Clinical and Experimental Obstetrics and Gynecology*, *42*(1), 49–52. https://pubmed.ncbi.nlm.nih.gov/25864281/

Chabrol, H., Teissedre, F., Saint-Jean, M., Teisseyre, N., Rogé, B., & Mullet, E. (2002). Prevention and treatment of post-partum depression: a controlled randomized study on women at risk. *Psychological Medicine*, *32*(6), 1039–1047. <https://doi.org/10.1017/s0033291702006062>

Chibanda, D., Shetty, A. K., Tshimanga, M., Woelk, G., Stranix-Chibanda, L., & Rusakaniko, S. (2014). Group problem-solving therapy for postnatal depression among HIV-positive and HIV-negative mothers in Zimbabwe. *Journal of the International Association of Providers of AIDS Care*, *13*(4), 335–341. <https://doi.org/10.1177/2325957413495564>

Cho, H. J., Kwon, J. H., & Lee, J. J. (2008). Antenatal cognitive-behavioral therapy for prevention of postpartum depression: A pilot study. *Yonsei Medical Journal*, *49*(4), 553–562. <https://doi.org/10.3349%2Fymj.2008.49.4.553>

Cinciripini, P. M., Blalock, J. A., Minnix, J. A., Robinson, J. D., Brown, V. L., Lam, C., … Karam-Hage, M. (2010). Effects of an intensive depression-focused intervention for smoking cessation in pregnancy. *Journal of Consulting and Clinical Psychology*, *78*(1), 44–54. <https://doi.org/10.1037/a0018168>

Cooper, P. J., Murray, L., Wilson, A., & Romaniuk, H. (2003). Controlled trial of the short- and long-term effect of psychological treatment of post-partum depression. I. Impact on maternal mood. *British Journal of Psychiatry*, *182*, 412–419. <https://doi.org/10.1192/bjp.02.177>

Dafei, M., Mojahed, S., Dastjerdi, G., Dehghani, A., & Shojaaddini Ardakani, T. (2021). The effect of cognitive–behavioral counseling of pregnant women with the presence of a spouse on stress, anxiety, and postpartum depression. *Journal of Education and Health Promotion*, *10*. <https://doi.org/10.4103%2Fjehp.jehp_926_20>

Danaher, B. G., Milgrom, J., Seeley, J. R., Stuart, S., Schembri, C., Tyler, M. S., … Lewinsohn, P. (2013). MomMoodBooster web-based intervention for postpartum depression: Feasibility trial results. *Journal of Medical Internet Research*, *15*(11), e242. <https://doi.org/10.2196/jmir.2876>

Dennis C. L. (2003). The effect of peer support on postpartum depression: A pilot randomized controlled trial. *Canadian Journal of Psychiatry*, *48*(2), 115–124. <https://doi.org/10.1177/070674370304800209>

Duffecy, J., Grekin, R., Hinkel, H., Gallivan, N., Nelson, G., & O'Hara, M. W. (2019). A group-based online intervention to prevent postpartum depression (Sunnyside): Feasibility randomized controlled trial. *JMIR Mental Health*, *6*(5). <https://doi.org/10.2196/10778>

Fonseca, A., Monteiro, F., Alves, S., Gorayeb, R., & Canavarro, M. C. (2019). Be a Mom, a web-based intervention to prevent postpartum depression: The enhancement of self-regulatory skills and its association with postpartum depressive symptoms. *Frontiers in Psychology*, *10*, 265. <https://doi.org/10.3389%2Ffpsyg.2019.00265>

Futterman, D., Shea, J., Besser, M., Stafford, S., Desmond, K., Comulada, W. S., & Greco, E. (2010). Mamekhaya: A pilot study combining a cognitive-behavioral intervention and mentor mothers with PMTCT services in South Africa. *AIDS Care*, *22*(9), 1093–1100. <https://doi.org/10.1080%2F09540121003600352>

George, C. (2020). Effectiveness of a group intervention led by lay health workers in reducing the incidence of postpartum depression in South India. *Asian Journal of Psychiatry, 47*, 101864. <https://doi.org/10.1016/j.ajp.2019.101864>

Ginsburg, G. S., Barlow, A., Goklish, N., Hastings, R., Baker, E. V., Mullany, B., … Walkup, J. (2012). Postpartum depression prevention for reservation-based American Indians: Results from a pilot randomized controlled trial. *Child and Youth Care Forum*, *41*(3), 229–245. <https://doi.org/10.1007/s10566-011-9161-7>

Goodman, J. H., Prager, J., Goldstein, R., & Freeman, M. (2015). Perinatal Dyadic Psychotherapy for postpartum depression: A randomized controlled pilot trial. *Archives of Women's Mental Health*, *18*(3), 493–506. <https://doi.org/10.1007/s00737-014-0483-y>

Gureje, O., Oladeji, B. D., Montgomery, A. A., Araya, R., Bello, T., Chisholm, D., … Zelkowitz, P. (2019). High- versus low-intensity interventions for perinatal depression delivered by non-specialist primary maternal care providers in Nigeria: Cluster randomised controlled trial (the EXPONATE trial). *British Journal of Psychiatry*, *215*(3):528-535. <https://doi.org/10.1192/bjp.2019.4>

Haga, S. M., Drozd, F., Lisøy, C., Wentzel-Larsen, T., & Slinning, K. (2019). Mamma Mia - A randomized controlled trial of an internet-based intervention for perinatal depression. *Psychological Medicine*, *49*(11), 1850–1858. <https://doi.org/10.1017/s0033291718002544>

Heller, H. M., Hoogendoorn, A. W., Honig, A., Broekman, B., & van Straten, A. (2020). The effectiveness of a guided internet-based tool for the treatment of depression and anxiety in pregnancy (MamaKits Online): Randomized controlled trial. *Journal of Medical Internet Research*, *22*(3), e15172. <https://doi.org/10.2196/15172>

Hou, Y., Hu, P., Zhang, Y., Lu, Q., Wang, D., Yin, L., … Zou, X. (2014). Cognitive behavioral therapy in combination with systemic family therapy improves mild to moderate postpartum depression. *Revista Brasileira de Psiquiatria*, *36*(1), 47–52. <https://doi.org/10.1590/1516-4446-2013-1170>

Howell, E. A., Balbierz, A., Wang, J., Parides, M., Zlotnick, C., & Leventhal, H. (2012). Reducing postpartum depressive symptoms among black and Latina mothers: A randomized controlled trial. *Obstetrics and Gynecology*, *119*(5), 942–949. <https://doi.org/10.1097/aog.0b013e318250ba48>

Howell, E. A., Bodnar-Deren, S., Balbierz, A., Loudon, H., Mora, P. A., Zlotnick, C., … & Leventhal, H. (2014). An intervention to reduce postpartum depressive symptoms: A randomized controlled trial. *Archives of Women's Mental Health*, *17*(1), 57–63. <https://doi.org/10.1007%2Fs00737-013-0381-8>

Husain, N., Zulqernain, F., Carter, L. A., Chaudhry, I. B., Fatima, B., Kiran, T., … Rahman, A. (2017). Treatment of maternal depression in urban slums of Karachi, Pakistan: A randomized controlled trial (RCT) of an integrated maternal psychological and early child development intervention. *Asian Journal of Psychiatry, 29*, 63-70. <https://doi.org/10.1016/j.ajp.2017.03.010>

Husain, N., Kiran, T., Fatima, B., Chaudhry, I., Husain, M., Shah, S., … Chaudhry, N. (2021). An integrated parenting intervention for maternal depression and child development in a low-resource setting: Cluster randomized controlled trial. *Depression and Anxiety, 38* (9), 925-939. <https://doi.org/10.1002/da.23169>

Jannati, N., Mazhari, S., Ahmadian, L., & Mirzaee, M. (2020). Effectiveness of an app-based cognitive behavioral therapy program for postpartum depression in primary care: A randomized controlled trial. *International Journal of Medical Informatics, 141*, 104145. <https://doi.org/10.1016/j.ijmedinf.2020.104145>

Jesse, D. E., Gaynes, B. N., Feldhousen, E. B., Newton, E. R., Bunch, S., & Hollon, S. D. (2015). Performance of a culturally tailored cognitive-behavioral intervention integrated in a public health setting to reduce risk of antepartum depression: A randomized controlled trial. *Journal of Midwifery & Women's Health*, *60*(5), 578–592. <https://doi.org/10.1111/jmwh.12308>

Jiang, L., Wang, Z. Z., Qiu, L. R., Wan, G. B., Lin, Y., & Wei, Z. (2014). Psychological intervention for postpartum depression. *Journal of Huazhong University of Science and Technology*, *34*(3), 437-442. <https://doi.org/10.1007/s11596-014-1297-x>

Kaaya, S. F., Blander, J., Antelman, G., Cyprian, F., Emmons, K. M., Matsumoto, K., … Smith Fawzi, M. C. (2013). Randomized controlled trial evaluating the effect of an interactive group counseling intervention for HIV-positive women on prenatal depression and disclosure of HIV status. *AIDS Care*, *25*(7), 854–862. <https://doi.org/10.1080/09540121.2013.763891>

Kim, D. R., Hantsoo, L., Thase, M. E., Sammel, M., & Epperson, C. N. (2014). Computer-assisted cognitive behavioral therapy for pregnant women with major depressive disorder. *Journal of Women's Health*, *23*(10), 842–848. <https://doi.org/10.1089%2Fjwh.2014.4867>

Kingston, D., Austin, M. P., Hegadoren, K., McDonald, S., Lasiuk, G., McDonald, S., … van Zanten, S. V. (2014). Study protocol for a randomized, controlled, superiority trial comparing the clinical and cost- effectiveness of integrated online mental health assessment-referral-care in pregnancy to usual prenatal care on prenatal and postnatal mental health and infant health and development: The Integrated Maternal Psychosocial Assessment to Care Trial (IMPACT). *Trials*, *15*, 72. <https://doi.org/10.1186/1745-6215-15-72>

Kozinszky, Z., Dudas, R. B., Devosa, I., Csatordai, S., Tóth, E., Szabó, D., … Pál, A. (2012). Can a brief antepartum preventive group intervention help reduce postpartum depressive symptomatology? *Psychotherapy and Psychosomatics*, *81*(2), 98–107. <https://doi.org/10.1159/000330035>

Le, H. N., Perry, D. F., & Stuart, E. A. (2011). Randomized controlled trial of a preventive intervention for perinatal depression in high-risk Latinas. *Journal of Consulting and Clinical Psychology*, *79*(2), 135–141. <https://doi.org/10.1037/a0022492>

Leung, S. S., & Lam, T. H. (2012). Group antenatal intervention to reduce perinatal stress and depressive symptoms related to intergenerational conflicts: A randomized controlled trial. *International Journal of Nursing Studies*, *49*(11), 1391–1402. <https://doi.org/10.1016/j.ijnurstu.2012.06.014>

Leung, S. S., Lee, A. M., Chiang, V. C., Lam, S. K., Kuen, Y. W., & Wong, D. F. (2013). Culturally sensitive, preventive antenatal group cognitive-behavioural therapy for Chinese women with depression. *International Journal of Nursing Practice*, *19 Suppl 1*, 28–37. <https://doi.org/10.1111/ijn.12021>

Leung, S. S., Lee, A. M., Wong, D. F., Wong, C. M., Leung, K. Y., Chiang, V. C., … Chung, K. F. (2016). A brief group intervention using a cognitive-behavioural approach to reduce postnatal depressive symptoms: A randomised controlled trial. *Hong Kong Medical Journal*, *22 Suppl 2*, S4–S8. <https://www.hkmj.org/system/files/hkm1602sp2p4.pdf>

Liu, H., & Yang, Y. (2021). Effects of a psychological nursing intervention on prevention of anxiety and depression in the postpartum period: A randomized controlled trial. *Annals of General Psychiatry, 20*(1), 2. <https://doi.org/10.1186/s12991-020-00320-4>

Loughnan, S. A., Sie, A., Hobbs, M. J., Joubert, A. E., Smith, J., Haskelberg, H., … Newby, J. M. (2019). A randomized controlled trial of 'MUMentum Pregnancy': Internet-delivered cognitive behavioral therapy program for antenatal anxiety and depression. *Journal of Affective Disorders*, *243*, 381–390. <https://doi.org/10.1016/j.jad.2018.09.057>

Lowndes, T. A., Egan, S. J., & McEvoy, P. M. (2019). Efficacy of brief guided self-help cognitive behavioral treatment for perfectionism in reducing perinatal depression and anxiety: A randomized controlled trial. *Cognitive Behaviour Therapy*, *48*(2), 106–120. <https://doi.org/10.1080/16506073.2018.1490810>

Mao, H. J., Li, H. J., Chiu, H., Chan, W. C., & Chen, S. L. (2012). Effectiveness of antenatal emotional self-management training program in prevention of postnatal depression in Chinese women. *Perspectives in Psychiatric Care*, *48*(4), 218–224. <https://doi.org/10.1111/j.1744-6163.2012.00331.x>

Mehri, M., Iravani, M., Bargard, M. S., & Haghighizadeh, M. H. (2019). Effectiveness of cognitive behavioral therapy-based self-management on depression in pregnant women: A randomized controlled trial. *Journal of Biochemical Technology, 10*(3): 92-97. <https://jbiochemtech.com/storage/models/article/uxPP64cmPM0MgAQkKTyJJCyEPc2Lokv8GRw7wwcYEaWMjRElmFgijy4vUm6Q/effectiveness-of-cognitive-behavioral-therapy-based-self-management-on-depression-in-pregnant-wome.pdf>

Milgrom, J., Schembri, C., Ericksen, J., Ross, J., & Gemmill, A. W. (2011a). Towards parenthood: An antenatal intervention to reduce depression, anxiety and parenting difficulties. *Journal of Affective Disorders*, *130*(3), 385–394. <https://doi.org/10.1016/j.jad.2010.10.045>

Muñoz, R. F., Le, H.-N., Ippen, C. G., Diaz, M. A., Urizar, G. G., Jr., Soto, J., … Lieberman, A. F. (2007). Prevention of postpartum depression in low-income women: Development of the Mamás y Bebés/ Mothers and Babies course. Cognitive and Behavioral Practice, 14(1), 70–83. <https://doi.org/10.1016/j.cbpra.2006.04.021>

Mureşan-Madar, A., & Băban, A. (2015). The development and piloting of a CBT group program for postpartum depression. *Journal of Evidence-Based Psychotherapies, 15*(1), 51–64. <https://www.proquest.com/docview/1672283828/fulltextPDF/8097985A715745FAPQ/1?accountid=14715>

Murray, L., Cooper, P. J., Wilson, A., & Romaniuk, H. (2003). Controlled trial of the short- and long-term effect of psychological treatment of post-partum depression: 2. Impact on the mother-child relationship and child outcome. *British Journal of Psychiatry, 182*, 420-427. <https://doi.org/10.1192/bjp.182.5.420>

Ngai, F. W., Wong, P. W. C., Chung, K. F., Chau, P. H., & Hui, P. W. (2020). Effect of couple-based cognitive behavioural intervention on prevention of postnatal depression: multisite randomised controlled trial. *BJOG: An International Journal of Obstetrics and Gynaecology*, 127(4), 500–507. <https://doi.org/10.1111/1471-0528.15862>

Nasiri, S., Kordi, M., & Gharavi, M. M. (2015). A comparative study of the effects of problem-solving skills training and relaxation on the score of self-esteem in women with postpartum depression. *Iranian Journal of Nursing and Midwifery Research*, *20*(1), 105–112. <https://pubmed.ncbi.nlm.nih.gov/25709699/>

Ortiz Collado, M. A., Saez, M., Favrod, J., & Hatem, M. (2014). Antenatal psychosomatic programming to reduce postpartum depression risk and improve childbirth outcomes: A randomized controlled trial in Spain and France. *BMC Pregnancy and Childbirth*, *14*, 22. <https://doi.org/10.1186/1471-2393-14-22>

Pinheiro, R. T., Botella, L., de Avila Quevedo, L., Amaral, K., Pinheiro, T, Jansen, K, … da Silva, R. A. (2014). Maintenance of the effects of cognitive behavioral and relational constructivist psychotherapies in the treatment of women with postpartum depression: A randomized clinical trial. *Journal of Constructivist Psychology, 27*(1), 59-68. <https://doi.org/10.1080/10720537.2013.814093>

Prendergast, J., & Austin, M.-P. (2001). Early childhood nurse-delivered cognitive behavioural counselling for post-natal depression. *Australasian Psychiatry, 9*(3), 255–259. <https://doi.org/10.1046%2Fj.1440-1665.2001.00330.x>

Puckering, C., McIntosh, E., Hickey, A., & Longford, J. (2010). Mellow Babies: A group intervention for infants and mothers experiencing postnatal depression. *Counselling Psychology Review, 25*(1), 28-40. <https://www.mellowparenting.org/wp-content/uploads/2016/03/Mellow-Babies-A-group-intervention-for-infants-and-mothers-PND-Puckering-et-al-2010.pdf>

Rahman, A., Malik, A., Sikander, S., Roberts, C., & Creed, F. (2008). Cognitive behaviour therapy-based intervention by community health workers for mothers with depression and their infants in rural Pakistan: A cluster-randomised controlled trial. *The Lancet,* *372*(9642), 902–909. <https://doi.org/10.1016/s0140-6736(08)61400-2>

Ramezani, S., Khosravi, A., Motaghi, Z., Hamidzadeh, A., & Mousavi, S. A. (2017). The effect of cognitive-behavioural and solution-focused counselling on prevention of postpartum depression in nulliparous pregnant women. *Journal of Reproductive and Infant Psychology*, *35*(2), 172–182. <https://doi.org/10.1080/02646838.2016.1266470>

Rees B. L. (1993). An exploratory study of the effectiveness of a relaxation with guided imagery protocol. *Journal of Holistic Nursing,* *11*(3), 271–276. <https://doi.org/10.1177/089801019301100306>

Sanders, M. R., & McFarland, M. (2000). Treatment of depressed mothers with disruptive children: A controlled evaluation of cognitive behavioral family intervention. Behavior Therapy, 31(1), 89–112. <https://doi.org/10.1016/S0005-7894(00)80006-4>

Sandner, M., Cornelissen, T., Jungmann, T., & Herrmann, P. (2018). Evaluating the effects of a targeted home visiting program on maternal and child health outcomes. *Journal of Health Economics*, *58*, 269–283. <https://doi.org/10.1016/j.jhealeco.2018.02.008>

Shamshiri Milani, H., Azargashb, E., Beyraghi, N., Defaie, S., & Asbaghi, T. (2015). Effect of telephone-based support on postpartum depression: A randomized controlled trial. *International Journal of Fertility and Sterility*, *9*(2), 247–253. <https://doi.org/10.22074%2Fijfs.2015.4246>

Sheeber, L. B., Seeley, J. R., Feil, E. G., Davis, B., Sorensen, E., Kosty, D. B., & Lewinsohn, P. M. (2012). Development and pilot evaluation of an Internet-facilitated cognitive-behavioral intervention for maternal depression. *Journal of Consulting and Clinical Psychology*, *80*(5), 739–749. <https://doi.org/10.1037/a0028820>

Silverstein, M., Diaz-Linhart, Y., Cabral, H., Beardslee, W., Broder-Fingert, S., Kistin, C. J., … Feinberg, E. (2018). Engaging mothers with depressive symptoms in care: Results of a randomized controlled trial in Head Start. *Psychiatric Services*, *69*(11), 1175–1180. <https://doi.org/10.1176/appi.ps.201800173>

Silverstein, M., Feinberg, E., Cabral, H., Linhart, Y. D., Sandler, J., Hegel, M., … Beardslee, W. (2011). Potential impact of trauma on the ability to prevent depression among low-income mothers. *Depression and Anxiety*, *28*(6), 478–484. <https://doi.org/10.1002/da.20817>

Stein, A., Netsi, E., Lawrence, P. J., Granger, C., Kempton, C., Craske, M. G., … Murray, L. (2018). Mitigating the effect of persistent postnatal depression on child outcomes through an intervention to treat depression and improve parenting: A randomised controlled trial. *The Lancet. Psychiatry*, *5*(2), 134–144. <https://doi.org/10.1016/S2215-0366(18)30006-3>

Surkan, P. J., Gottlieb, B. R., McCormick, M. C., Hunt, A., & Peterson, K. E. (2012). Impact of a health promotion intervention on maternal depressive symptoms at 15 months postpartum. *Maternal and Child Health Journal*, *16*(1), 139–148. <https://doi.org/10.1007/s10995-010-0729-x>

Tandon, S. D., Leis, J. A., Mendelson, T., Perry, D. F., & Kemp, K. (2014). Six-month outcomes from a randomized controlled trial to prevent perinatal depression in low-income home visiting clients. *Maternal and Child Health Journal*, *18*(4), 873–881. <https://doi.org/10.1007/s10995-013-1313-y>

Tandon, S. D., Perry, D. F., Mendelson, T., Kemp, K., & Leis, J. A. (2011). Preventing perinatal depression in low-income home visiting clients: A randomized controlled trial. *Journal of Consulting and Clinical Psychology*, *79*(5), 707–712. <https://doi.org/10.1037/a0024895>

Tandon, S. D., Ward, E. A., Hamil, J. L., Jimenez, C., & Carter, M. (2018). Perinatal depression prevention through home visitation: A cluster randomized trial of mothers and babies 1-on-1. *Journal of Behavioral Medicine*, *41*(5), 641–652. <https://doi.org/10.1007/s10865-018-9934-7>

Ugarriza D. N. (2004). Group therapy and its barriers for women suffering from postpartum depression. *Archives of Psychiatric Nursing*, *18*(2), 39–48. <https://doi.org/10.1053/j.apnu.2004.01.002>

Ugarriza, D. N., & Schmidt, L. (2006). Telecare for women with postpartum depression. *Journal of Psychosocial Nursing and Mental Health Services*, *44*(1), 37–45. <https://doi.org/10.3928/02793695-20060101-08>

Van Lieshout, R. J., Layton, H., Feller, A., Ferro, M. A., Biscaro, A., & Bieling, P. J. (2020). Public health nurse delivered group cognitive behavioral therapy (CBT) for postpartum depression: A pilot study. *Public Health Nursing*, *37*(1), 50–55. <https://doi.org/10.1111/phn.12664>

Van Lieshout, R. J., Yang, L., Haber, E., & Ferro, M. A. (2017). Evaluating the effectiveness of a brief group cognitive behavioural therapy intervention for perinatal depression. *Archives of Women's Mental Health*, *20*(1), 225–228. <https://doi.org/10.1007/s00737-016-0666-9>

Van Ravesteyn, L. M., Kamperman, A. M., Schneider, T., Raats, M. E., Steegers, E., Tiemeier, H., … & Lambregtse-van den Berg, M. P. (2018). Group-based multicomponent treatment to reduce depressive symptoms in women with co-morbid psychiatric and psychosocial problems during pregnancy: A randomized controlled trial. *Journal of Affective Disorders*, *226*, 36–44. <https://doi.org/10.1016/j.jad.2017.09.019>

Yazdanimehr, R., Omidi, A., Sadat, Z., & Akbari, H. (2016). The effect of mindfulness-integrated cognitive behavior therapy on depression and anxiety among pregnant women: A randomized clinical trial. *Journal of Caring Sciences*, *5*(3), 195–204. <https://doi.org/10.15171%2Fjcs.2016.021>
