# Supplementary material for: Effectiveness of cognitive behavioural therapy-based interventions for maternal perinatal depression: a systematic review and meta-analysis
Source: BMC Psychiatry. 2023 Mar 29;23:208. doi: 10.1186/s12888-023-04547-9 (PMC10052839; doi:10.1186/s12888-023-04547-9)
Supplement: Supplementary file 10 — Additional file 10. References for included studies. [file 12888_2023_4547_MOESM10_ESM.docx]

**S10. References for included studies**

Alhusen, J. L., Hayat, M. J., & Borg, L. (2021). A pilot study of a group-based perinatal depression intervention on reducing depressive symptoms and improving maternal-fetal attachment and maternal sensitivity. *Archives of Women’s Mental Health, 24*(1), 145–154. <https://doi.org/10.1007/s00737-020-01032-0>

Ammerman, R. T., Putnam, F. W., Altaye, M., Stevens, J., Teeters, A. R., & Van Ginkel, J. B. (2013). A clinical trial of in-home CBT for depressed mothers in home visitation. *Behavior Therapy, 44*(3), 359–372. <https://doi.org/10.1016/j.beth.2013.01.002>

Burns, A., O’Mahen, H., Baxter, H., Bennert, K., Wiles, N., Ramchandani, P., … Evans, J. (2013). A pilot randomised controlled trial of cognitive behavioural therapy for antenatal depression. *BMC Psychiatry, 13*, 33. <https://doi.org/10.1186/1471-244X-13-33>

Dimidjian, S., Goodman, S. H., Sherwood, N. E., Simon, G. E., Ludman, E., Gallop, R., … Beck, A. (2017). A pragmatic randomized clinical trial of behavioral activation for depressed pregnant women. *Journal of Consulting and Clinical Psychology, 85*(1), 26–36. <https://doi.org/10.1037/ccp0000151>

Forsell, E., Bendix, M., Hollandare, F., von Schultz, B. S., Nasiell, J., Blomdahl-Wetterholm, M., … Kaldo, V. (2017). Internet delivered cognitive behavior therapy for antenatal depression: A randomised controlled trial. *Journal of Affective Disorders, 221*, 56–64. <https://doi.org/10.1186/1471-244X-13-33>

Fuhr, D. C., Weobong, B., Lazarus, A., Vanobberghen, F., Weiss, H. A., Singla, D. R., … Patel, V. (2019). Delivering the Thinking Healthy Programme for perinatal depression through peers: an individually randomised controlled trial in India. *The Lancet Psychiatry, 6*(2), 115–127. <https://doi.org/10.1016/S2215-0366(18)30466-8>

Honey, K. L., Bennett, P., & Morgan, M. (2002). A brief psycho-educational group intervention for postnatal depression. *British Journal of Clinical Psychology, 41*(4), 405–409. <https://doi.org/10.1348/014466502760387515>

Hughes, M. et al. (2015). Report on the NFN Depression Improvement Study: A Clinical Trial Testing In-Home CBT. Office of Early Childhood, Family Support Services, Center for Social Research, University of Hartford.

Khamseh, F., Parandeh, A., Hajiamini, Z., Tadrissi, S. D., & Najjar, M. (2019). Effectiveness of applying problem‑solving training on depression in Iranian pregnant women: Randomized clinical trial. *Journal of Education and Health Promotion, 8*, 87. <https://doi.org/10.4103/jehp.jehp>

Lund, C., Schneider, M., Garman, E. C., Davies, T., Munodawafa, M., Honikman, S., … Susser, E. (2020). Task-sharing of psychological treatment for antenatal depression in Khayelitsha, South Africa: Effects on antenatal and postnatal outcomes in an individual randomised controlled trial. *Behaviour Research and Therapy, 130*, 103466. <https://doi.org/10.1016/j.brat.2019.103466>

*McKee, M. D., Zayas, L. H., Fletcher, J., Boyd, R. C., & Nam, S. H. (2006). Results of an intervention to reduce perinatal depression among low-income minority women in community primary care. *Journal of Social Service Research, 32*(4), 63–81. <https://doi.org/10.1300/J079v32n04_04>

*Meager, I., & Milgrom, J. (1996). Group treatment for postpartum depression: A pilot study. *Australian and New Zealand Journal of Psychiatry, 30*(6), 852–860. <https://doi.org/10.3109/00048679609065055>

Milgrom, J., Danaher, B. G., Gemmill, A. W., Holt, C. J. C. J., Holt, C. J. C. J., Seeley, J. R., … Ericksen, J. (2016). Internet cognitive behavioral therapy for women with postnatal depression: A randomized controlled trial of MumMoodBooster. *Journal of Medical Internet Research, 18*(3), 1–18. <https://doi.org/10.2196/jmir.4993>

Milgrom, J., Gemmill, A. W., Ericksen, J., Burrows, G., Buist, A., & Reece, J. (2015). Treatment of postnatal depression with cognitive behavioural therapy, sertraline and combination therapy: A randomised controlled trial. *Australian and New Zealand Journal of Psychiatry, 49*(3), 236–245. <https://doi.org/10.1177/0004867414565474>

Milgrom, J., Holt, C., Holt, C. J., Ross, J., Ericksen, J., & Gemmill, A. W. (2015). Feasibility study and pilot randomised trial of an antenatal depression treatment with infant follow-up. *Archives of Women’s Mental Health, 18*(5), 717–730. <https://doi.org/10.1007/s00737-015-0512-5>

Milgrom, J., Holt, C. J., Gemmill, A. W., Ericksen, J., Leigh, B., Buist, A., & Schembri, C. (2011). Treating postnatal depressive symptoms in primary care: A randomised controlled trial of GP management, with and without adjunctive counselling. *BMC Psychiatry*, 11, 19 <https://doi.org/10.1186/1471-244X-11-95>

Milgrom, J., Negri, L. M., Gemmill, A. W., McNeil, M., Martin, P. R., J., M., … Martin, P. R. (2005). A randomized controlled trial of psychological interventions for postnatal depression. *British Journal of Clinical Psychology, 44*(4), 529–542. <https://doi.org/10.1348/014466505X34200>

Misri, S., Reebye, P., Corral, M., Mills, L., & Milis, L. (2004). The use of paroxetine and cognitive-behavioral therapy in postpartum depression and anxiety: A randomized controlled trial*. Journal of Clinical Psychiatry, 65*(9), 1236–1241. <https://doi.org/10.4088/JCP.v65n0913>

Morrell, C. J., Slade, P., Warner, R., Paley, G., Dixon, S., Walters, S. J., … Nicholl, J. (2009). Clinical effectiveness of health visitor training in psychologically informed approaches for depression in postnatal women: Pragmatic cluster randomised trial in primary care. *British Medical Journal*, 338, a3045. <https://doi.org/10.1136/bmj.a3045>

Nasiri, S., Kordi, M., Gharavi, M. M., & Lotfabadi, M. K. (2018). Effect of problem-solving therapy and relaxation on the severity of postpartum depressive symptoms: A randomized controlled trial. *Nursing and Midwifery Studies, 7*(1), 6–11. <https://doi.org/10.4103/nms.nms_35_17>

*Ngai, F. W., Wong, P. W., Leung, K. Y., Chau, P. H., & Chung, K. F. (2015). The effect of telephone-based cognitive-behavioral therapy on postnatal depression: A randomized controlled trial. *Psychotherapy and Psychosomatics*, 84(5), 294–303. <https://doi.org/10.1159/000430449>

O’Mahen, H., Woodford, J., McGinley, J., Warren, F. C., Richards, D. A., Lynch, T. R., & Taylor, R. S. (2013). Internet-based behavioral activation-Treatment for postnatal depression (Netmums): A randomized controlled trial. *Journal of Affective Disorders, 150*(3), 814–822. <https://doi.org/10.1016/j.jad.2013.03.005>

O'Mahen, H., Himle, J. A., Fedock, G., Henshaw, E., & Flynn, H. (2013). A pilot randomized controlled trial of cognitive behavioral therapy for perinatal depression adapted for women with low incomes. *Depression and Anxiety, 30*(7), 679–687. <https://doi.org/10.1002/da.22050>

O'Mahen, H. A., Richards, D. A., Woodford, J., Wilkinson, E., McGinley, J., Taylor, R. S., & Warren, F. C. (2014). Netmums: a phase II randomized controlled trial of a guided Internet behavioural activation treatment for postpartum depression. *Psychological Medicine*, 44(8), 1675–1689. <https://doi.org/10.1017/s0033291713002092>

Pugh, N. E., Hadjistavropoulos, H. D., & Dirkse, D. (2016). A randomised controlled trial of Therapist-Assisted, Internet-delivered cognitive behavior therapy for women with maternal depression. *PLoS ONE, 11*(3), 1–13. <https://doi.org/10.1371/journal.pone.0149186>

Rojas, G., Fritsch, R., Solis, J., Jadresic, E., Castillo, C., González, M., … Araya, R. (2007). Treatment of postnatal depression in low-income mothers in primary-care clinics in Santiago, Chile: A randomised controlled trial. *Lancet, 370*(9599), 1629–1637. <https://doi.org/10.1016/S0140-6736(07)61685-7>

Sikander, S., Ahmad, I., Atif, N., Zaidi, A., Vanobberghen, F., Weiss, H. A., … Rahman, A. (2019). Delivering the Thinking Healthy Programme for perinatal depression through volunteer peers: A cluster randomised controlled trial in Pakistan. *The Lancet Psychiatry, 6*(2), 128–139. <https://doi.org/10.1016/S2215-0366(18)30467-X>

Trevillion, K., Ryan, E. G., Pickles, A., Heslin, M., Byford, S., Nath, S., … Howard, L. M. (2020). An exploratory parallel-group randomised controlled trial of antenatal Guided Self-Help (plus usual care) versus usual care alone for pregnant women with depression: DAWN trial. *Journal of Affective Disorders, 261*, 187–197. <https://doi.org/10.1016/j.jad.2019.10.013>

Van Lieshout, R. J., Layton, H., Savoy, C. D., Brown, J. S. L., Ferro, M. A., Streiner, D. L., Bieling, P. J., Feller, A., & Hanna, S. (2021). Effect of online 1-day cognitive behavioral therapy-based workshops plus usual care vs usual care alone for postpartum depression: A randomized clinical trial. *JAMA Psychiatry, 78*(11), 1200–1207. <https://doi.org/10.1001/jamapsychiatry.2021.2488>

*Wiklund, I., Mohlkert, P., & Edman, G. (2010). Evaluation of a brief cognitive intervention in patients with signs of postnatal depression: a randomized controlled trial. *Acta Obstetricia et Gynecologica Scandinavica, 89*(8), 1100–1104. <https://doi.org/10.3109/00016349.2010.500369>

*Wozney, L., Olthuis, J., Lingley-Pottie, P., McGrath, P. J., Chaplin, W., Elgar, F., … Kennedy, J. (2017). Strongest Families (TM) Managing Our Mood (MOM): A randomized controlled trial of a distance intervention for women with postpartum depression. *Archives of Women’s Mental Health, 20*(4), 525–537. <https://doi.org/10.1007/s00737-017-0732-y>

**denotes included in systematic review only*
