# Supplementary material for: Effectiveness of cognitive behavioural therapy-based interventions for maternal perinatal depression: a systematic review and meta-analysis
Source: BMC Psychiatry. 2023 Mar 29;23:208. doi: 10.1186/s12888-023-04547-9 (PMC10052839; doi:10.1186/s12888-023-04547-9)
Supplement: Supplementary file 11 — Additional file 11. Severity of depression calculation. [file 12888_2023_4547_MOESM11_ESM.docx]

**S11. Severity of depression calculation**

| **Study ID** | **Author** | **Year** | **Intervention** | **Time point** | **Outcome measure** | **IV 1N** | **IV Baseline DEP** | **Control N** | **Control Baseline DEP** | **IV 2 N** | **IV 2 Basline DEP** | **Overall Baseline DEP** | **Severity of depression at baseline** |
| --- | --- | --- | --- | --- | --- | --- | --- | --- | --- | --- | --- | --- | --- |
| 168 | Alhusen | 2021 | CBT | Baseline | EPDS | 30 | 17.8 | 30 | 17.4 |  |  | 17.6 | Moderate |
| 9 | Ammerman | 2013 | CBT | Baseline | EPDS | 47 | 18.77 | 46 | 19.22 |  |  | 18.9925806 | Severe |
| 43 | Burns | 2013 | CBT | Baseline | EPDS | 18 | 17.56 | 18 | 19.17 |  |  | 18.365 | Moderate |
| 44 | Dimidjian | 2017 | BA | Baseline | PHQ-9 | 86 | 14.86 | 77 | 14.6 |  |  | 14.7371779 | Moderate |
| 70 | Forsell | 2017 | CBT | Baseline | MADRS-S | 22 | 24.2 | 20 | 24.4 |  |  | 24.2952381 | Moderate |
| 161 | Fuhr | 2019 | CBT | Baseline | PHQ-9 | 140 | 13.66 | 140 | 13.09 |  |  | 13.375 | Moderate |
| 144 | Honey | 2002 | CBT | Baseline | EPDS | 23 | 19.35 | 22 | 17.95 |  |  | 18.6655556 | Moderate |
| 129 | Hughs | 2015 | CBT | Baseline | EPDS | 41 | 18.23 | 29 | 16.1 |  |  | 17.35 | Moderate |
| 120 | Khamseh | 2019 | Problem solving | Baseline | BDI-II | 35 | 16.06 | 35 | 15.34 |  |  | 15.7 | Mild |
| 170 | Lund | 2020 | Problem solving and BA | Baseline | HDRS | 184 | 15.7 | 200 | 15.5 |  |  | 15.5958333 | Mild |
| 123 | McKee | 2006 | CBT | Baseline | BDI-II | 57 | 20.5 | 43 | 22.4 |  |  | 21.317 | Moderate |
| 58 | Meager | 1996 | CBT | Baseline | EPDS | 10 | 24.8 | 10 | 27.5 |  |  | 26.15 | Severe |
| 49 | Milgrom | 2005 | CBT | Baseline | BDI-II | 46 | 22.96 | 33 | 20.12 |  |  | 21.7736709 | Moderate |
| 107 | Milgrom | 2011 | CBT | Baseline | BDI-II | 22 | 25.5 | 23 | 27.9 | 23 | 30.9 | 28.1382353 | Moderate |
| 56 | Milgrom | 2015 A | CBT | Baseline | BDI-II | 27 | 30.7 | 27 | 30.77 |  |  | 30.735 | Severe |
| 113 | Milgrom | 2015 B | CBT | Baseline | BDI-II | 15 | 27.53 | 16 | 31.94 |  |  | 29.806129 | Severe |
| 69 | Milgrom | 2016 | CBT | Baseline | BDI-II | 21 | 25.3 | 22 | 26.3 |  |  | 25.8116279 | Moderate |
| 147 | Misri | 2004 | CBT | Baseline | EPDS | 15 | 18.87 | 13 | 18.15 |  |  | 18.5357143 | Moderate |
| 26 | Nasiri | 2018 | Problem solving | Baseline | BDI-II (Persian) | 40 | 19.27 | 40 | 20 |  |  | 19.635 | Moderate |
| 19 | O'Mahen | 2013 A | CBT | Baseline | BDI-II | 30 | 29.93 | 25 | 26.56 |  |  | 28.3981818 | Moderate |
| 71 | O'Mahen | 2013 B | BA | Baseline | EPDS | 462 | 19.46 | 448 | 19.44 |  |  | 19.4501538 | Moderate |
| 79 | O'Mahen | 2014 | BA | Baseline | EPDS | 41 | 20.24 | 42 | 21.07 |  |  | 20.66 | Severe |
| 45 | Pugh | 2016 | CBT | Baseline | EPDS | 25 | 14.92 | 25 | 15.13 |  |  | 15.025 | Moderate |
| 124 | Rojas | 2007 | CBT | Baseline | EPDS | 114 | 17.7 | 116 | 17.1 |  |  | 17.3973913 | Moderate |
| 160 | Sikander | 2019 | CBT | Baseline | PHQ-9 | 283 | 14.89 | 287 | 14.48 |  |  | 14.6835614 | Moderate |
| 173 | Lieshout | 2021 | CBT | Baseline | EPDS | 202 | 16.47 | 201 | 15.92 |  |  | 16.19568238 | Moderate |
| 40 | Wiklund | 2010 | CBT | Baseline | EPDS (Swedish) | 33 | 16.9 | 34 | 13.6 |  |  | 15.2253731 | Moderate |
| 100 | Wozney | 2017 | CBT | Baseline | EPDS | 32 | 16.34 | 30 | 16.18 |  |  | 16.2625806 | Moderate |
