# Supplementary material for: Effectiveness of cognitive behavioural therapy-based interventions for maternal perinatal depression: a systematic review and meta-analysis
Source: BMC Psychiatry. 2023 Mar 29;23:208. doi: 10.1186/s12888-023-04547-9 (PMC10052839; doi:10.1186/s12888-023-04547-9)
Supplement: Supplementary file 12 — Additional file 12. Depression outcome cut offs. [file 12888_2023_4547_MOESM12_ESM.docx]

S12. Depression outcome cut offs

| **Outcome measure** | **Cut offs** | **Reference** |
| --- | --- | --- |
| EPDS | 0-6 none or minimal depression  7-13 mild depression 14-19 moderate depression  19-30 severe depression | McCabe-Beane, J.E., Segre, L.S., Perkhounkova, Y., Stuart, S., & O'Hara, M.W. (2016). The identification of severity ranges for the Edinburgh Postnatal Depression Scale. Journal of Reproductive and Infant Psychology, 34, (3). |
| PHQ-9 | 0-5 mild 6-10 moderate 11-15 moderately severe 16-20 severe depression | Kroenke, K., Spitzer, R.L., & Williams, J.B. (2001). The PHQ-9: Validity of a Brief Depression Severity Measure. Journal of General Internal Medicine. 16 (9). |
| BDI-II | 0–13 minimal depression 14–19 mild depression 20–28 moderate depression 29–63 severe depression | Beck, A.T., Steer, R.A. & Garbin, M.G. (1988). Psychometric properties of the Beck Depression Inventory Twenty-five years of evaluation. Clinical Psychological Review (8). |
| HDRS | 0-7 no depression  8-16 mild depression  17-23 moderate depression  ≥24 severe depression | Zimmerman, M., Martinez, J.H., Young, D., Chelminski, I. & Dalrymple, K. (2013). Severity classification on the Hamilton depression rating scale. Journal of Affective Disorders 15 (2). |
| MADRS-S | 0 to 6 normal/symptom absent 7 to 19 mild depression 20 to 34 moderate depression >34 severe depression | Montgomery-Åsberg Depression Rating Scale (MADRS) in BioPsychoSocial Assessment Tools for the Elderly - Assessment Summary Sheet. The University of Western Ontario, London, Ontario, Canada |
