# Supplementary figures and images for: Effectiveness of cognitive behavioural therapy-based interventions for maternal perinatal depression: a systematic review and meta-analysis
Source: BMC Psychiatry. 2023 Mar 29;23:208. doi: 10.1186/s12888-023-04547-9 (PMC10052839; doi:10.1186/s12888-023-04547-9)

**S13. Prediction interval**


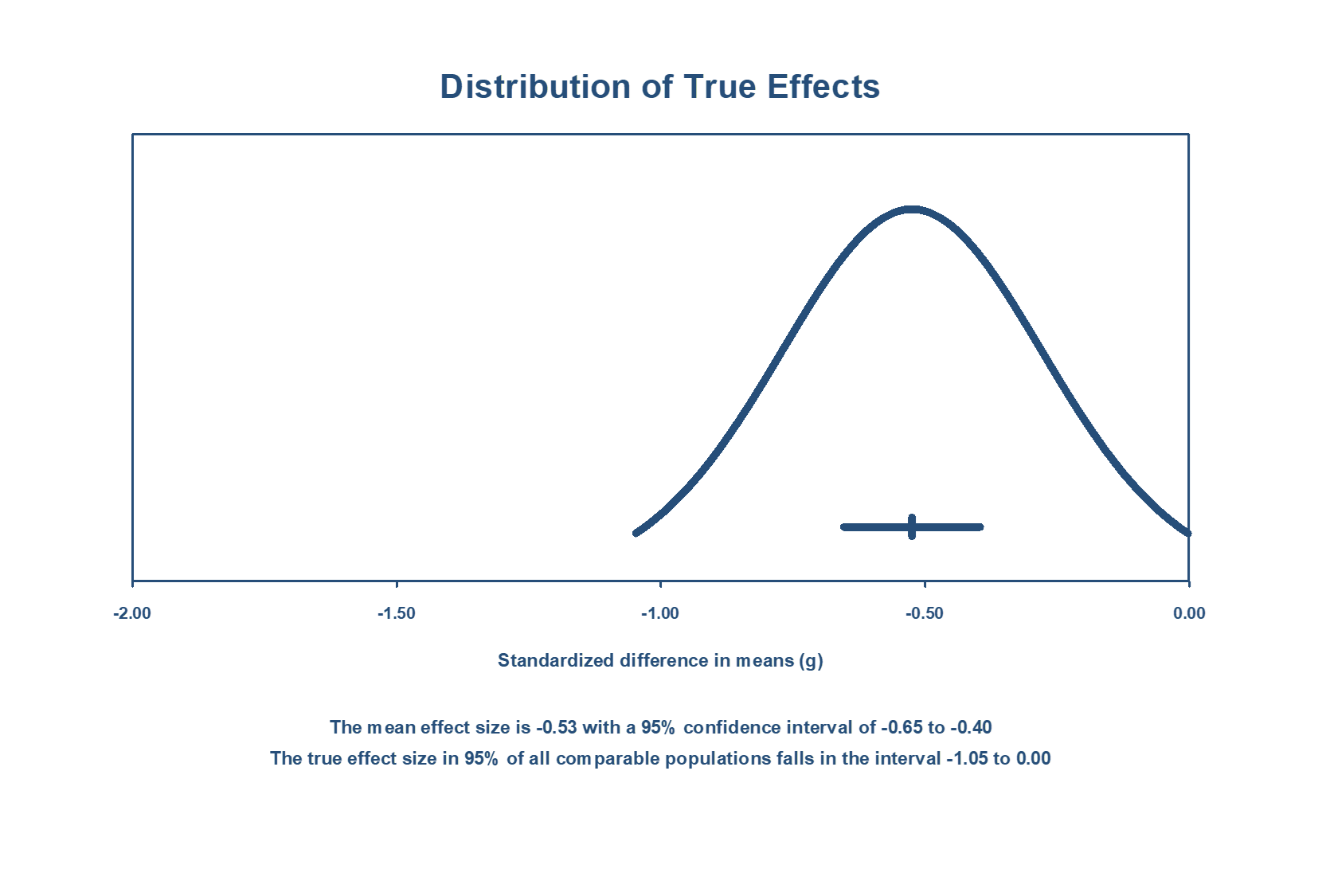

Supplement: Supplementary file 13 — Additional file 13. Prediction interval. [file 12888_2023_4547_MOESM13_ESM.docx]

**S16. Funnel plot for anxiety**


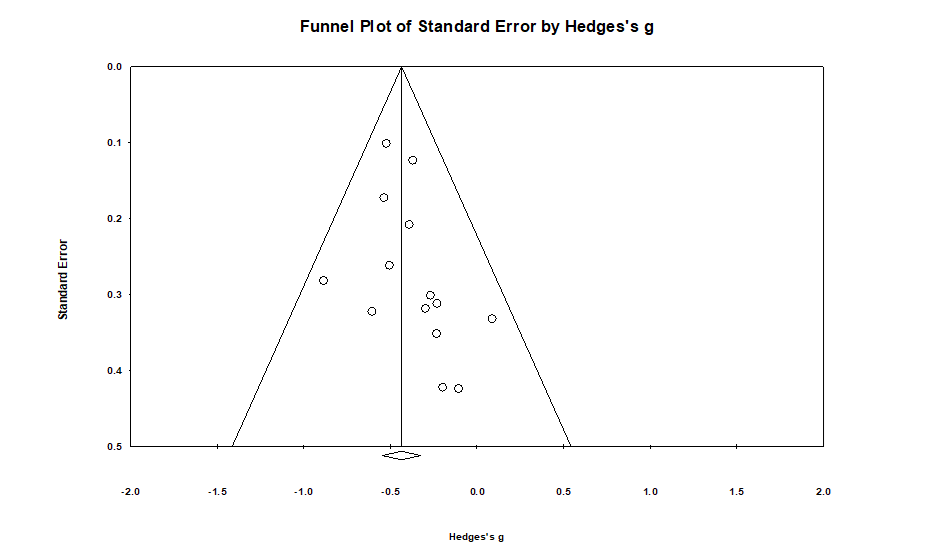

Supplement: Supplementary file 16 — Additional file 16. Risk of bias assessment. [file 12888_2023_4547_MOESM16_ESM.docx]
