# Supplementary material for: Effectiveness of cognitive behavioural therapy-based interventions for maternal perinatal depression: a systematic review and meta-analysis
Source: BMC Psychiatry. 2023 Mar 29;23:208. doi: 10.1186/s12888-023-04547-9 (PMC10052839; doi:10.1186/s12888-023-04547-9)
Supplement: Supplementary file 15 — Additional file 15. Funnel plot for anxiety. [file 12888_2023_4547_MOESM15_ESM.docx]

**S15. Risk of bias assessment**

| Study | Randomization | Data collection method | Randomization process | Deviations from intended interventions | Missing outcome data | Measurement of the outcome | Selection of the reported result | Overall |
| --- | --- | --- | --- | --- | --- | --- | --- | --- |
| Alhusen et al., (2021) | Individual | Per protocol | **+** | **+** | **+** | **!** | **!** | **!** |
| Ammerman et al., (2013) | Individual | ITT | **+** | **+** | **+** | **!** | **+** | **!** |
| Burns et al., (2013) | Individual | Per protocol | **+** | **+** | **+** | **!** | **+** | **!** |
| Dimidjan et al., (2017) | Individual | Per protocol | **+** | **+** | **+** | **!** | **+** | **!** |
| Forsell et al., (2017) | Individual | Per protocol | **!** | **+** | **+** | **!** | **+** | **!** |
| Fuhr et al.,  (2019) | Individual | ITT | **+** | **+** | **+** | **!** | **+** | **!** |
| Honey et al., (2002) | Individual | ITT | **!** | **+** | **+** | **!** | **+** | **!** |
| Hughs et al., (2015) | Individual | ITT | **!** | **+** | **+** | **!** | **+** | **!** |
| Khamseh et al., (2019) | Individual | Per protocol | **!** | **+** | **+** | **!** | **+** | **!** |
| Lund et al.,  (2020) | Individual | ITT | **+** | **+** | **+** | **!** | **+** | **!** |
| Milgrom et al., (2005) | Individual | ITT | **+** | **+** | **+** | **!** | **+** | **!** |
| Milgrom et al., (2011) | Individual | ITT | **!** | **+** | **+** | **!** | **!** | **!** |
| Milgrom et al., (2015a) | Individual | ITT | **!** | **+** | **+** | **!** | **+** | **!** |
| Milgrom et al,. (2015b) | Individual | ITT | **!** | **+** | **+** | **!** | **+** | **!** |
| Milgrom et al., (2016) | Individual | ITT | **+** | **+** | **+** | **!** | **+** | **!** |
| Misri et al.,  (2004) | Individual | ITT | **!** | **!** | **+** | **!** | **+** | **!** |
| Morell et al., (2009) | Cluster | ITT | **+** | **+** | **!** | **-** | **+** | **-** |
| Nasiri et al., (2018) | Individual | Per protocol | **!** | **+** | **+** | **!** | **+** | **!** |
| O´Mahen et al., (2013a) | Individual | Per protocol | **+** | **+** | **+** | **!** | **+** | **!** |
| O´Mahen et al., (2013b) | Individual | ITT | **+** | **+** | **+** | **!** | **+** | **!** |
| O´Mahen et al., (2014) | Individual | Per protocol | **+** | **+** | **+** | **!** | **+** | **!** |
| Pugh et al.,  (2016) | Individual | ITT | **+** | **+** | **+** | **!** | **+** | **!** |
| Rojas et al., (2007) | Individual | Per protocol | **+** | **+** | **+** | **!** | **+** | **!** |
| Sikander et al., (2019) | Cluster | ITT | **+** | **+** | **!** | **!** | **+** | **!** |
| Van Lieshout (2021) | Individual | ITT | **+** | **+** | **+** | **!** | **+** | **!** |

| **+** | Low risk |
| --- | --- |
| **!** | Some concerns |
| **-** | High risk |
