# Supplementary material for: Effectiveness of cognitive behavioural therapy-based interventions for maternal perinatal depression: a systematic review and meta-analysis
Source: BMC Psychiatry. 2023 Mar 29;23:208. doi: 10.1186/s12888-023-04547-9 (PMC10052839; doi:10.1186/s12888-023-04547-9)
Supplement: Supplementary file 4 — Additional file 4. Peer review assessment form. [file 12888_2023_4547_MOESM4_ESM.docx]

S4. Peer review assessment form

**PRESS Guideline — Search Submission & Peer Review Assessment**

**SEARCH SUBMISSION: *THIS SECTION TO BE FILLED IN BY THE SEARCHER***

**Searcher:** Danelle Pettman **Email:** [Danelle.pettman@kbh.uu.se](mailto:Danelle.pettman@kbh.uu.se)

**Reviewer:** Professor Alkistis Skalkidou

**Date submitted:** 12.11.2018 **Date requested by**: 19.11.2018

**Systematic Review Title:**

Effectiveness and acceptability of cognitive behavioural therapy based interventions for maternal peripartum depression: A systematic review, meta-analysis and thematic synthesis.

This search strategy is …

| X | My PRIMARY (core) database strategy — First time submitting a strategy for search question and database |
| --- | --- |
|  | My PRIMARY (core) strategy — Follow-up review NOT the first time submitting a strategy for search question and database. If this is a response to peer review, itemize the changes made to the review suggestions |
|  | SECONDARY search strategy— First time submitting a strategy for search question and database |
|  | SECONDARY search strategy — NOT the first time submitting a strategy for search question and database. If  this is a response to peer review, itemize the changes made to the review suggestions |

**Database**

(i.e., MEDLINE,CINAHL…):	*[mandatory]*

Medline

**Interface**

(i.e., Ovid, EBSCO…):	*[mandatory]*

Pubmed

**Additional database searches**

*Participants.* Adult women with either a diagnosis of PDD, or reporting some level of depression symptomatology with a peripartum onset using a validated tool (e.g., Edinburgh Postnatal Depression Scale (EPDS).

*Interventions.* CBT, behavioural activation (BA) and problem-solving interventions explicitly targeting an improvement in peripartum depression as a primary outcome.

*Comparators.* All control conditions will be considered.

*Outcomes:* Primary outcomes of interest are self-report, clinician or proxy administered standardised measurement of depression or peripartum depession. Secondary outcomes of interest are standardised measures of (a) anxiety; (b) stress; (c) parent-infant attachment; (d) maternal sensitivity; (e) perceived social support; and (f) parental competence. Qualitative outcomes. Qualitative data reporting participants experience and perspectives of treatment, including satisfaction, relevance and suggested adaptations to improve acceptability.

*Study design.* Only randomized control trials will be included.

Once the primary search strategy is confirmed we will also search; ISI Web of Science; Cumulative Index to Nursing and Allied Health Literature (CINAHL); Cochrane Central Register of Controlled Trials (CENTRAL); Prospero; Excerpta Medica DataBase (EMBASE); Applied Social Sciences Index and Abstracts (ASSIA); Psychinfo; SCOPUS; and SweMed+

**Research Question**

(Describe the purpose of the search)	*[mandatory]*

(a) to examine the overall effectiveness of CBT based interventions for peripartum depression; (b) to examine the effectiveness of CBT based interventions on secondary outcomes; (c) to investigate clinical and methodological moderators associated with effectiveness; (d) to describe mothers’ perceptions of the acceptability of CBT based interventions for peripartum, and (e) to identify adaptations to CBT based interventions associated with acceptability

**PICO Format**

(Outline the PICOs for your question — i.e., Patient, Intervention, Comparison, Outcome, and Study Design — as applicable)

| **P** | Mothers from conception to 1 year postpartum suffering with depression or low mood |
| --- | --- |
| **I** | All variants of behavioural, cognitive and cognitive-behavioural therapy |
| **C** | All comparators will be considered |
| **O** | Measures of depression or peripartum depression |
| **S** | Randomised control trials |

**Inclusion Criteria** (List criteria such as age groups, study designs, etc., to be included) *[optional]*

**Exclusion Criteria**

(List criteria such as study designs, date limits, etc., to be excluded) *[optional]*

*Participants.* (a) the treatment of mood disorders other than depression (e.g. bipolar affective disorder); and (b) interventions focused on prevention of maternal psychopathology in at-risk, but not currently symptomatic mothers.

Only studies in English or Swedish will be included.

**Was a search filter applied?**

No	Yes

**If YES, which one(s) (e.g., Cochrane RCT filter, PubMed Clinical Queries filter)? Provide the source if this is a published filter.** *[mandatory if YES to previous question* — *textbox]*

Other notes or comments you feel would be useful for the peer reviewer? ***[optional]***

We had considered not including the concept 4 therapy terms, however this reduces the specificity of the search.

Please copy and paste your search strategy here, exactly as run, including the number of hits per line. ***[mandatory]***

| **Concept 1** | **Concept 2** | **Concept 3** | **Concept 4** | **Concept 5** |
| --- | --- | --- | --- | --- |
| **Perinatal** | **Depression terms** | **CBT** | **Therapy terms** | **RCT** |
| **Title & Abstract**  Postpartum 47725  post-partum 11003  antepartum 5374  ante-partum 423  partum 12020  prepartum 2077  pre-partum 329  intrapartum 7905  intra-partum 319  peripartum 4151  peri-partum 160  postnatal 96485  post-natal 6733  Perinatal 65465  peri-natal 177  antenatal 31533  ante-natal 511  prenatal 87822  pre-natal 1028  pregnan* 480609  puerper* 14147  maternal 231206  trimester 49419  impregnated 10808  gravid* 12698  multigravid* 1185  primigravid* 4766  parity 29634  obstetric 39258  gestation 109623  before birth 3679  in utero 25721  **Mesh terms**  Pregnancy 844017  postpartum period 59467  prenatal care 24767  perinatal care 8957  care, postnatal 5085  pregnancy complications 400382  depression, postpartum 4780 | **Title & Abstract**  depress* 416497  depressive 102129  low mood 621  mood 66046  distress 99553  wellbeing 12032  well-being 63718  emotion 33746  emotional 133450  melanchol* 2906  affect 589721  affective 51447  affective symptoms 1647  negative affect 8508  dysphori*5153  affective disorder 6830  **Mesh terms**  affect 30820  mood disorders 112524  depression 195007  depressive disorder 100595  affective symptoms 12245  affective disorder 112524 | **Title & Abstract**  cognitive 306140  cognitive behavio* 23129  behavio* 1122596  CBT 9128  BA 25470  problem solving 16422  ccbt 155  icbt 551  cognitive restructuring 785  cognitive reframing 84  activity scheduling 39  **Mesh terms**  behavior therapy 67509  cognitive therapy 24435  behavior therapies, cognitive 24435 | **Title & Abstract**  Therapy 1749047  Therapies 248514  Psychotherapy 35578  Intervention 515758  Management 983110  program evaluation 6170  program 400451  programs 255468  programme 86763  programmes 55208  group 2335915  course 515471  online 86832  Internet 45003  Web 89875  web-based 25325  Phone 18507  Telephone 52303  Skype 273  e-therapy 452  etherapy 9  computer-assisted 24439  computerized 67756  internet intervention* 462  computer* 281642  mobile 85365  tablet* 48173  smartphone* 6968  internet-administered 65  ementalhealth 26  e-mental health 175  m-mental health 3  Mmentalhealth 1  Ehealth 2629  e-health 2356 | **Publication type**  Randomized controlled trial 470367  systematic review 222096  Clinical trial 809548  controlled clinical trial 558223  meta-analysis 93273  **Title & Abstract**  Randomized controlled trial 57325  Randomized control trial 3210  RCT 18011  Controlled 684318  randomi* 557922  random assignment 2191  random allocation 1516  random 239928  randomly 299405  control 2340336  controls 775814  feasibility 154608  pilot 132357  comparative study 72223  follow up 852728  meta-analysis 116966  metaanalysis 1531  Review 1392398  **Mesh terms**  allocation, random 96464  randomized controlled trial 121644  meta analysis 17054  randomized controlled trials as topic 121644  meta analysis as topic 17054  review, systematic 0 |
| **Total for concept 1: 1230102** | **Total for concept 2: 1670493** | **Total for concept 3:**  **1414869** | **Total for concept 4: 6226286** | **Total for concept 5:**  **6135718** |
|  |  |  |  | **Total for all: 3917** |

**Syntax:**

(((((((((((((((((((((((((((((((((((((postpartum[Title/Abstract]) OR post-partum antepartum[Title/Abstract]) OR ante-partum partum[Title/Abstract]) OR prepartum[Title/Abstract]) OR pre-partum[Title/Abstract]) OR intrapartum[Title/Abstract]) OR intra-partum[Title/Abstract]) OR peripartum[Title/Abstract]) OR peri-partum[Title/Abstract]) OR postnatal[Title/Abstract]) OR post-natal[Title/Abstract]) OR Perinatal[Title/Abstract]) OR peri-natal[Title/Abstract]) OR antenatal[Title/Abstract]) OR ante-natal[Title/Abstract]) OR prenatal[Title/Abstract]) OR pre-natal[Title/Abstract]) OR pregnan*[Title/Abstract]) OR puerper[Title/Abstract]) OR puerper*[Title/Abstract]) OR maternal[Title/Abstract]) OR trimester[Title/Abstract]) OR impregnated[Title/Abstract]) OR gravid*[Title/Abstract]) OR multigravid*[Title/Abstract]) OR primigravid*[Title/Abstract]) OR parity[Title/Abstract]) OR obstetric[Title/Abstract]) OR gestation[Title/Abstract]) OR before birth[Title/Abstract]) OR in utero[Title/Abstract])) OR (((((((pregnancy[MeSH Terms]) OR postpartum period[MeSH Terms]) OR prenatal care[MeSH Terms]) OR perinatal care[MeSH Terms]) OR care, postnatal[MeSH Terms]) OR pregnancy complications[MeSH Terms]) OR depression, postpartum[MeSH Terms]))) AND (((((((((((((((((((depress*[Title/Abstract]) OR depressive[Title/Abstract]) OR low mood[Title/Abstract]) OR mood[Title/Abstract]) OR distress[Title/Abstract]) OR wellbeing[Title/Abstract]) OR well being[Title/Abstract]) OR emotion[Title/Abstract]) OR emotional[Title/Abstract]) OR melanchol*[Title/Abstract]) OR affect[Title/Abstract]) OR affective[Title/Abstract]) OR affective symptoms[Title/Abstract]) OR negative affect[Title/Abstract]) OR dysphori*[Title/Abstract]) OR affective disorder[Title/Abstract])) OR ((((((affect[MeSH Terms]) OR mood disorders[MeSH Terms]) OR depression[MeSH Terms]) OR depressive disorder[MeSH Terms]) OR affective symptoms[MeSH Terms]) OR affective disorder[MeSH Terms]))) AND (((((((((((((cognitive[Title/Abstract]) OR cognitive behavio*[Title/Abstract]) OR behavio*[Title/Abstract]) OR CBT[Title/Abstract]) OR BA[Title/Abstract]) OR problem solving[Title/Abstract]) OR ccbt[Title/Abstract]) OR icbt[Title/Abstract]) OR cognitive restructuring[Title/Abstract]) OR cognitive reframing[Title/Abstract]) OR activity scheduling[Title/Abstract])) OR (((cognitive behavior therapy[MeSH Terms]) OR behavior therapy[MeSH Terms]) OR cognitive therapy[MeSH Terms]))) AND (((((((((((((((((((((((((((((((((((therapy[Title/Abstract]) OR therapies[Title/Abstract]) OR psychotherapy[Title/Abstract]) OR intervention[Title/Abstract]) OR management[Title/Abstract]) OR program evaluation[Title/Abstract]) OR program[Title/Abstract]) OR programs[Title/Abstract]) OR programme[Title/Abstract]) OR programmes[Title/Abstract]) OR group[Title/Abstract]) OR course[Title/Abstract]) OR online[Title/Abstract]) OR Internet[Title/Abstract]) OR Web[Title/Abstract]) OR web-based[Title/Abstract]) OR Phone[Title/Abstract]) OR Telephone[Title/Abstract]) OR Skype[Title/Abstract]) OR e-therapy[Title/Abstract]) OR Etherapy[Title/Abstract]) OR computer-assisted[Title/Abstract]) OR computerized[Title/Abstract]) OR internet intervention*[Title/Abstract]) OR computer*[Title/Abstract]) OR mobile[Title/Abstract]) OR tablet*[Title/Abstract]) OR smartphone*[Title/Abstract]) OR internet-administered[Title/Abstract]) OR ementalhealth[Title/Abstract]) OR e-mental health[Title/Abstract]) OR m-mental health[Title/Abstract]) OR Mmentalhealth[Title/Abstract]) OR Ehealth[Title/Abstract]) OR e-health[Title/Abstract])) AND (((((((Randomized controlled trial[Publication Type]) OR systematic review[Publication Type]) OR controlled clinical trial[Publication Type]) OR meta-analysis[Publication Type])) OR ((((((((((((((((((Randomized controlled trial[Title/Abstract]) OR Randomized control trial[Title/Abstract]) OR RCT[Title/Abstract]) OR Controlled[Title/Abstract]) OR randomi*[Title/Abstract]) OR random assignment[Title/Abstract]) OR random allocation[Title/Abstract]) OR random[Title/Abstract]) OR randomly[Title/Abstract]) OR control[Title/Abstract]) OR controls[Title/Abstract]) OR feasibility[Title/Abstract]) OR pilot[Title/Abstract]) OR comparative study[Title/Abstract]) OR follow up[Title/Abstract]) OR meta-analysis[Title/Abstract]) OR metaanalysis[Title/Abstract]) OR Review[Title/Abstract])) OR ((((((allocation, random[MeSH Terms]) OR randomized controlled trial[MeSH Terms]) OR meta analysis[MeSH Terms]) OR randomized controlled trials as topic[MeSH Terms]) OR meta analysis as topic[MeSH Terms]) OR review, systematic[MeSH Terms]))

**(Add more space, as necessary.)**

**PEER REVIEW ASSESSMENT: *THIS SECTION TO BE FILLED IN BY THE REVIEWER***

**Reviewer:** Professor Alkistis Skalkidou

Date completed: 12/12/2018

|  | **1. TRANSLATION** |  |  | | |
| --- | --- | --- | --- | --- | --- |
|  | | A -­‐No revisions | | ☐ |  |
|  | | B -­‐ Revision(s) suggested | | ☐ |  |
|  | | C -­‐ Revision(s) required | | ☐ |  |

If “B” or “C,” please provide an explanation or example:

*Does the search strategy match the research question/PICO?*

Yes

*Are the search concepts clear?*

Yes

*Are there too many or too few PICO elements included?*

Adequate- but I wonder if the algorithm will work well when you require CBT AND therapy- will it catch something that only has CBT in the text? The word CBT includes the concept of therapy

Same goes for MESH term depression, postpartum AND depression…

*Are the search concepts too narrow or too broad?*

Ok but sometimes they include each other, see above- Maybe this is not a problem but should be checked.

*Does the search retrieve too many or too few records?* (Please show number of hits per line.)

As expected

*Are unconventional or complex strategies explained?*

Yes

**2. BOOLEAN AND PROXIMITY OPERATORS**

| A -­‐No revisions | ☐ |
| --- | --- |
| B -­‐ Revision(s) suggested | ☐ |
| C -­‐ Revision(s) required | ☐ |

If “B” or “C,” please provide an explanation or example:

*Are Boolean or proximity operators used correctly?*

Yes

*Is the use of nesting with brackets appropriate and effective for the search?*

Yes

*If NOT is used, is this likely to result in any unintended exclusions?*

-

*Could precision be improved by using proximity operators (eg, adjacent, near, within) or phrase searching instead of AND?*

Probably, should be checked

*Is the width of proximity operators suitable (eg, might adj5 pick up more variants than adj2)?*

Not used

**3. SUBJECT HEADINGS**

| A -­‐No revisions | ☐ |
| --- | --- |
| B -­‐ Revision(s) suggested | ☐ |
| C -­‐ Revision(s) required | ☐ |

If “B” or “C,” please provide an explanation or example:

*Are the subject headings relevant?*

Yes

*Are any subject headings too broad or too narrow?*

No but somethings they intercept

*Are subject headings exploded where necessary and vice versa?*

Yes

*Are major headings (“starring” or restrict to focus) used? If so, is there adequate justification?*

-

*Are subheadings missing?*

No

*Are subheadings attached to subject headings? (Floating subheadings may be preferred.)*

Are not

*Are floating subheadings relevant and used appropriately?*

Have not seen any used (Subheading [sh:noexp])

*Are both subject headings and terms in free text (see the following) used for each concept?*

Yes

**4. TEXT WORD SEARCHING**

| A -­‐No revisions | ☐ |
| --- | --- |
| B -­‐ Revision(s)suggested | ☐ |
| C -­‐ Revision(s) required | ☐ |

If “B” or “C,” please provide an explanation or example:

*Does the search include all spelling variants in free text (eg, UK vs. US spelling)?*

Yes

*Does the search include all synonyms or antonyms (eg, opposites)?*

Perinatal concept include: “Maternity”, “Partus” and “Obstetrical”

Depression concept include: “dysthymia” and “alexithymia”

*Does the search capture relevant truncation (ie, is truncation at the correct place)?*

Yes

*Is the truncation too broad or too narrow?*

Adequate

*Are acronyms or abbreviations used appropriately? Do they capture irrelevant material? Are the full terms also included?*

Yes

*Have the appropriate fields been searched; for example, is the choice of the text word fields (.tw.) or all fields (.af.) appropriate? Are there any other fields to be included or excluded (database specific)?*

OK

Should any long strings be broken into several shorter search statements?

-

**5. SPELLING, SYNTAX, AND LINE NUMBERS**

| A -­‐No revisions | ☐ |
| --- | --- |
| B -­‐ Revision(s)suggested | ☐ |
| C -­‐ Revision(s) required | ☐ |

If “B” or “C,” please provide an explanation or example:

*Are there any spelling errors?*

I have not identified any.

*Are there any errors in system syntax; for example, the use of a truncation symbol from a different search interface?*

No

*Are there incorrect line combinations or orphan lines (ie, lines that are not referred to in the final summation that could indicate an error in an AND or OR statement)?*

No

**6. LIMITS AND FILTERS**

| A -­‐No revisions | ☐ |
| --- | --- |
| B -­‐ Revision(s) suggested | ☐ |
| C -­‐ Revision(s) required | ☐ |

If “B” or “C,” please provide an explanation or example:

OVERALL EVALUATION (Note: If one or more “revision required” is noted above, the response below must be “revisions required”.)

| A -­‐No revisions | ☐ |
| --- | --- |
| B -­‐ Revision(s) suggested | ☐ |
| C -­‐ Revision(s) required | ☐ |

Additional comments:

Some more comments- look at my review on ART and endometrial cancer, Cochrane, on how we looked at grey literature (additional databases).

I also thought that you have the whole perinatal period as one, and it could be very different effects of interventions for those depressed during and/or after pregnancy- I do not know if you want to already now plan for sensitivity analyses according to timing of intervention and/or maternal depression. Outcome has the same problem- at which time is depression measured? Just a thought.
